# Two Key Ferredoxins for Nitrogen Fixation Have Different Specificities and Biophysical Properties

**DOI:** 10.1002/chem.202500844

**Published:** 2025-05-30

**Authors:** Holly Addison, Pascal Pfister, Ana Lago‐Maciel, Tobias J. Erb, Antonio J. Pierik, Johannes G. Rebelein

**Affiliations:** ^1^ Microbial Metalloenzymes Research Group Max Planck Institute for Terrestrial Microbiology Marburg 35043 Germany; ^2^ Biochemistry and Synthetic Metabolism Research Group Max Planck Institute for Terrestrial Microbiology Marburg 35043 Germany; ^3^ Centre for Synthetic Microbiology (SYNMIKRO) Philipps University Marburg Marburg 35043 Germany; ^4^ Biochemistry, Department of Chemistry RPTU Kaiserslautern‐Landau Kaiserslautern 67663 Germany

**Keywords:** electron transport, ferredoxin, metalloenzyme, nitrogenase, nitrogen fixation

## Abstract

Ferredoxins deliver electrons to drive many challenging biochemical transformations, including enzyme‐catalyzed nitrogen fixation. We recently showed two distinct ferredoxins, FdC and FdN, were essential for iron nitrogenase‐mediated nitrogen fixation in *R. capsulatus*. In this study, we perform investigations on FdC and FdN to establish their key differences in terms of specificity, structure, and electronic properties. In vivo complementation studies of both the genes encoding FdC (*fdxC*) and FdN (*fdxN*), into ∆*fdxC* and ∆*fdxN R. capsulatus*‐deletion strains under N_2_‐fixing conditions, showed that plasmid‐based expression of *fdxN* recovered diazotrophic growth and Fe‐nitrogenase activity in both ∆*fdxC* and ∆*fdxN* strains, while plasmid‐based *fdxC* expression could only complement the ∆*fdxC* strain. Spectroscopic analysis of FdC and FdN using electron paramagnetic resonance spectroscopy revealed large differences in the electronic features of FdC and FdN. These differences were accompanied by large structural differences between FdC and FdN, assessed by a crystallographic structure of FdC and an AlphaFold model of FdN. We report novel features in the FdC structure, in terms of secondary structure and hydrogen‐bonding network, compared with structures of other [Fe_2_S_2_]‐cluster ferredoxins. Overall, we explore the biophysical properties that influence ferredoxin specificity, while providing new insights into the properties of ferredoxins essential for N_2_‐fixation.

## Graphical abstract

Two ferredoxins, FdC and FdN, are collectively essential for the process of nitrogen fixation by the iron nitrogenase in *R. capsulatus*. We explore the biophysical factors, through spectroscopic and structural characterizations, which determine ferredoxin functionality and pathway specificity. The structure of FdC (PDB: 9I2A) and model of FdN (AF‐D5ARY6) are shown, nitrogen fixation by the iron nitrogenase is depicted (PDB: 8OIE).

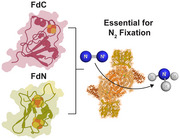

## 1 Introduction

Electron transfer events are an essential part of cellular biochemistry and are indispensable for sustaining biological life. This transfer of electrons from one cofactor to another is characterized by both the gain of an electron, called a reduction event, and the loss of an electron, called an oxidation event. Most famously, the core metabolic pathways of oxygenic photosynthesis and aerobic respiration are comprised of sequential electron transfer events involving complex electron‐transfer chains, connected by both cellular redox coenzymes and small electron transfer proteins.^[^ ^1^ ^]^

**Figure 1:**
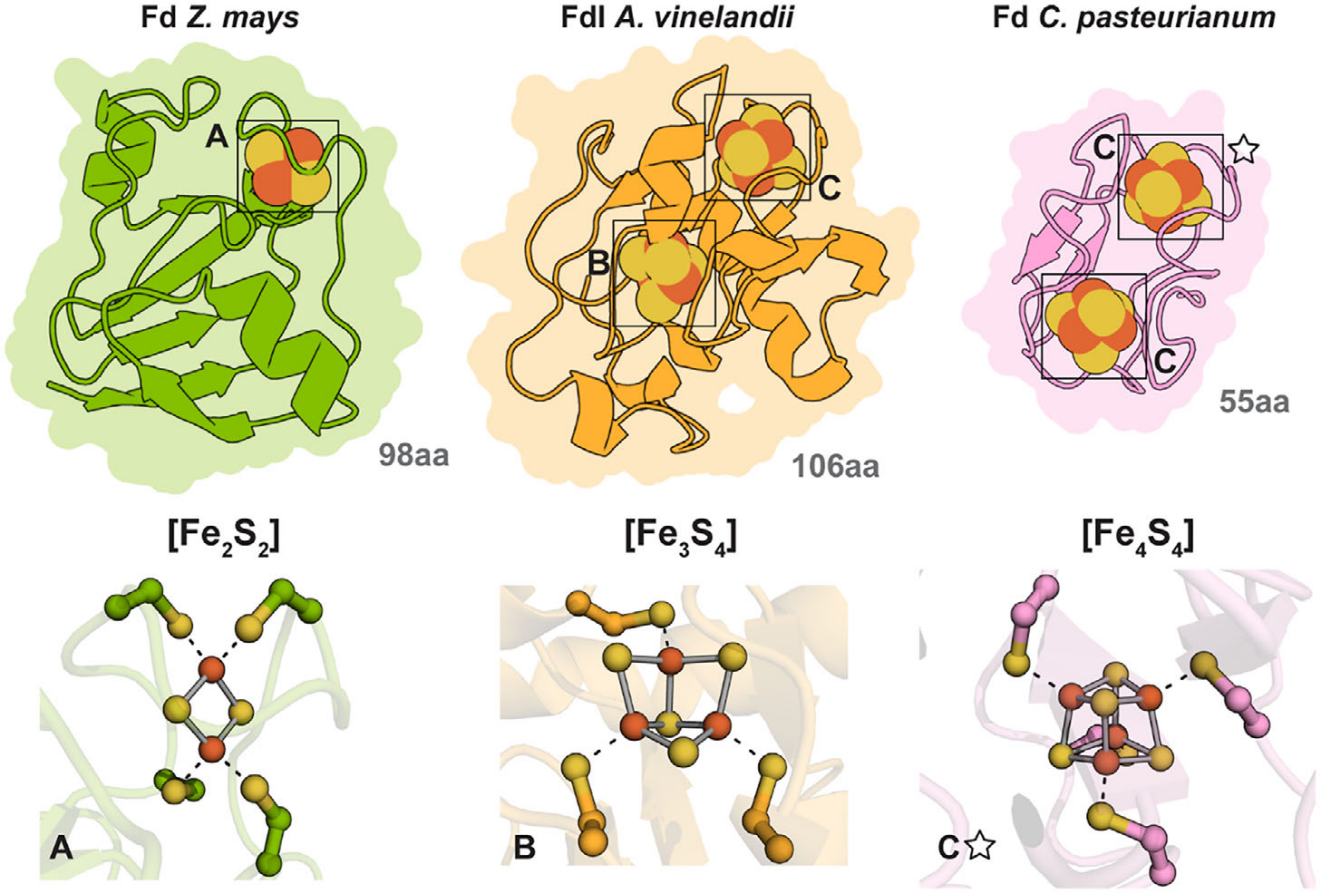
Ferredoxins are a structurally diverse group of enzymes. Fd structures and corresponding FeS‐clusters of Fd from *Z. mays* (left, green, PDB: 3B2F^[^ ^4^ ^]^), FdI from *A. vinelandii* (middle, orange, PDB: 7FD1^[^ ^5^ ^]^) and Fd from *C. pasteurianum* (right, pink, PDB: 1CLF^[^ ^6^ ^]^). **(Top)** Structures of ferredoxins (Fds) that coordinate different types of FeS‐clusters. Fd structures are shown as overlaid surface and cartoon representations. Labelled boxes match the labeling for the FeS‐cluster types below. The star icon is used to indicate which [Fe_4_S_4_]‐cluster is visualized on the bottom right. The amino acid (aa) sequence length of the individual Fds is shown in grey below. **(Bottom)** FeS‐cluster types and coordinating cysteine residues visualized as ball‐and‐stick representations. Iron atoms are colored in orange and sulfur atoms in yellow.

Some of the most well studied and diverse small electron transfer proteins bind cofactors called iron‐sulfur (FeS)‐clusters. A FeS‐cluster is a cofactor that comprises chemically bonded positively charged iron (Fe^2+/3+^) ions and sulfide ions (S^2−^) in different configurations. The most well characterized FeS‐cluster types are [Fe_2_S_2_]‐, [Fe_3_S_4_]‐, and [Fe_4_S_4_]‐clusters (Figure 1). Studies have shown that under specific early Earth conditions FeS‐clusters can form spontaneously, though within cells FeS‐clusters are enzymatically assembled.^[^ ^2^, ^3^ ^]^

Small electron transport proteins that bind FeS‐clusters are called ferredoxins (Fds). Within Fds, the FeS‐clusters are typically coordinated via cysteine (Cys) residues. Despite sharing a common name, there are many differences between individual Fds. Fds vary in size, oligomeric state, as well as the type and number of FeS‐clusters they contain (Figure 1). These structural differences influence the biophysical properties of different Fds, and most importantly, the overall reduction potential of Fds.^[^ ^7^ ^]^

Due to the large range of different properties between distinct Fds, they are involved in many essential metabolic processes such photosynthesis and oxidative phosphorylation. The selected Fds shown in Figure 1 are not only diverse in structure but also in cellular functions. Firstly, Fd isolated from maize (*Zea mays*) leaves contains a [Fe_2_S_2_]‐cluster and is essential for oxygenic photosynthesis. *Zea mays* Fd has been shown to interact with ferredoxin‐NADP^+^ reductase (FNR) to shuttle electrons from photosystem I to FNR to produce NADPH, which is utilized for CO_2_ fixation by the Calvin–Benson Cycle.^[^ ^8^, ^9^ ^]^ Additionally, *Z. mays* Fd is also known to interact with both nitrite and sulfite reductases for the processes of nitrogen and sulfur assimilation.^[^ ^4^, ^10^ ^]^ On the other hand, FdI from *Azotobacter vinelandii* is structurally different, binding one [Fe_3_S_4_]‐cluster and one [Fe_4_S_4_]‐cluster, and is believed to be involved in nitrogen fixation.^[^ ^11^ ^]^ *Clostridium pasteurianum* Fd differs again, binding two [Fe_4_S_4_]‐clusters and playing a role in anaerobic carbon metabolism and hydrogen uptake, acting as an electron acceptor for both pyruvate ferredoxin oxidoreductase and hydrogenase.^[^ ^12^, ^13^ ^]^

Fd also play a role in biological nitrogen fixation, whereby atmospheric nitrogen (N_2_) is selectively reduced to bioavailable ammonia (NH_3_) by enzymes called nitrogenases. Nitrogenases catalyze N_2_ reduction using energy from the hydrolysis of ATP and low potential electrons donated by small electron carriers specifically Fds or flavodoxins (Flds), the latter of which uses flavin mononucleotide instead of FeS‐clusters as the redox cofactor for electron transfer.^[^ ^14^ ^]^ The optimal stoichiometry for the reduction of N_2_ by the canonical molybdenum (Mo)‐nitrogenase is shown in equation (1)^[^ ^15^ ^]^:

(1) $$N_{2}+8H^{+}+16\;\mathrm{MgATP}+8e^{-}\to 2\;{\mathrm{NH}}_{3}+H_{2}+16\mathrm{MgADP}+16\;P_{i}$$

Beyond the Mo‐nitrogenase, there are two alternative nitrogenase isoforms: the vanadium (V)‐ and iron (Fe)‐nitrogenases. Each nitrogenase isoform is classified based on the identity of the heteroatom in their active site cofactors: the iron‐molybdenum, iron‐vanadium and iron‐iron cofactors (FeMoco, FeVco, FeFeco, respectively). The different structures and cofactors between the nitrogenase isoforms influence their efficiencies and specificities for a range of gaseous substrates, including carbon dioxide, carbon monoxide and acetylene.^[^ ^16^ ^]^

Within several diazotrophic (N_2_‐fixing) organisms, such as *Azotobacter vinelandii, R. capsulatus*, and *Rhodopseudomonas palustris*, electron carriers essential for Mo‐nitrogenase based nitrogen fixation have been identified and characterized.^[^ ^14^ ^]^ Until recently, it was not known which Fds or Flds are essential for N_2_‐fixation by the Fe‐nitrogenase. In the model diazotroph *R. capsulatus*, we showed that two distinct Fds are essential for N_2_‐fixation by the Fe‐nitrogenase and that the two Fds likely have different roles.^[^ ^17^ ^]^ One of the two Fds, a two [Fe_4_S_4_]‐cluster binding Fd called FdN, has high phylogenetic similarities to Fds from other organisms known to act as electron donors to the Mo‐nitrogenase, with these Fds specifically transferring electrons to the homodimeric reductase component of the Mo‐nitrogenase. Due to high sequence similarity between the reductases of the nitrogenase isoforms, it is hypothesized that FdN can also donate electrons to the Fe‐nitrogenase reductase component (AnfH_2_). Initial studies of FdN inferred the presence of two [Fe_4_S_4_]‐clusters from the sequence and electron paramagnetic resonance spectroscopy (EPR).^[^ ^18^, ^19^, ^20^ ^]^ The reduction potentials of both [Fe_4_S_4_]‐clusters within FdN were previously determined to be −490 mV (all potentials refer to the normal hydrogen electrode).^[^ ^18^ ^]^ Despite these characterizations, the unique nature of the EPR spectra for FdN still raises questions as to the electronic properties of FdN. Further, it is believed that FdN interacts with the *Rhodobacter* nitrogen fixation (Rnf) complex, a membrane‐integrated electron transport complex that provides electrons for N_2_‐fixation, which is proposed to employ energy from an electrochemical gradient to reduce FdN (E_m_ = −490 mV) using NADH (E_m_ = −320 mV).^[^ ^21^ ^]^ The other Fd, a [Fe_2_S_2_]‐cluster binding Fd called FdC, could not be easily classified despite containing the conserved [Fe_2_S_2_]‐cluster coordination motif (CX_4_CX_2_CX_n_C) typical for “plant‐type” Fds.^[^ ^22^, ^23^ ^]^ We discovered that FdC is the first [Fe_2_S_2_]‐cluster Fd involved in N_2_‐fixation under anaerobic conditions.^[^ ^17^ ^]^ FdC was shown to interact with an oxidoreductase of unknown function called FprA, encoded within the same operon as the gene for FdC (*fdxC*).^[^ ^24^ ^]^ FprA belongs to a family of flavoproteins, called A‐type flavoproteins, which includes F_420_H_2_ oxidase and nitric oxide reductase.^[^ ^25^, ^26^, ^27^ ^]^ However, an in depth spectroscopic and structural analysis of this protein is lacking.

In this study, we spectroscopically and structurally characterize FdC and FdN. Firstly, we observed different specificities for FdC and FdN in vivo using complementation studies under N_2_‐fixing conditions. It was shown that the *fdxN* gene could recover diazotrophic growth in both ∆*fdxN* and ∆*fdxC* strains, while *fdxC* could only recover growth in the ∆*fdxC* strain. These results suggested key differences in the properties of FdC and FdN, despite our finding that both Fds have key roles in maintaining N_2_‐fixation.^[^ ^17^ ^]^ Hence, both FdC and FdN were purified for in vitro characterization. FdC and FdN were isolated with their respective FeS‐clusters intact, meaning no cluster reconstitution was necessary. Fd characterization, via anaerobic UV‐Vis spectroscopy and electron paramagnetic resonance (EPR) spectroscopy, revealed key differences in the properties of both Fds. Additionally, here we report the high‐resolution (1.7 Å) structure of *R. capsulatus* FdC, the first structure of a [Fe_2_S_2_]‐cluster binding Fd involved in N_2_‐fixation under anaerobic conditions. The structure revealed key structural differences between the *R. capsulatus* FdC and other plant‐type [Fe_2_S_2_]‐cluster binding Fds. Our findings provide new insights into Fd properties and specificities, as well as establishing FdC as a novel class of [Fe_2_S_2_]‐cluster binding Fd essential for N_2_‐fixation in diazotrophic phototrophs.

## 2 Results

### 2.1 FdC and FdN Have Different In Vivo Specificities

**Figure 2:**
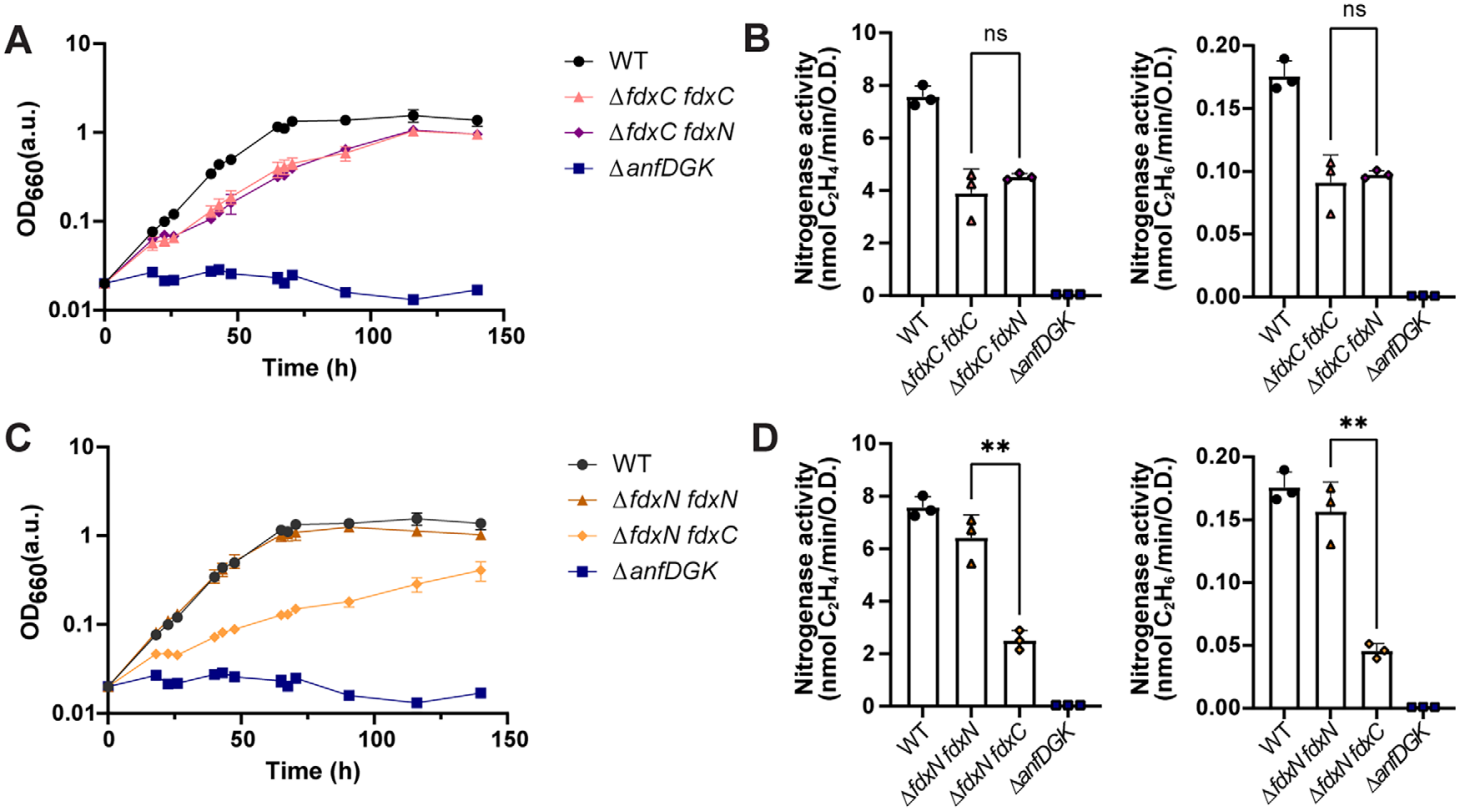
*fdxN* complements a ∆*fdxC R. capsulatus* strain while *fdxC* cannot complement a ∆*fdxN R. capsulatus* strain. (A, C) Fe‐nitrogenase dependent diazotrophic growth of (A) ∆*fdxC R. capsulatus* strains, (C) ∆*fdxN R. capsulatus* strains. (B, D) In vivo Fe‐nitrogenase activity determined by acetylene reduction assays for (B) ∆*fdxC R. capsulatus* strains, (D) ∆*fdxN R. capsulatus* strains. An unpaired *t*‐test with a 95% confidence level was used to determine significance in Fe‐nitrogenase activity between different strains. “ns” stands for “not significant” and “**” indicated a significant difference, with a P‐value < 0.0021, between two groups. (A‐D) The use of “∆” before an italicized gene name indicates this gene has been deleted from the genome of the *R. capsulatus* strain. A complemented gene expressed from a plasmid, within a *R. capsulatus* strain, is indicated by the italicized gene name. All *R. capsulatus* strains have deletions of ∆*nifD* and ∆*modABC* to promote the expression of the Fe‐nitrogenase genes (*anf*). The negative control ∆*anfDGK R. capsulatus* strain has the genes encoding the Fe‐nitrogenase catalytic component deleted. The optical density (OD) at 660 nm reports on cell growth, as the turbidity is proportional to the number of cells per volume.

Previously, we showed that the deletion of both *fdxC* and *fdxN* genes (∆*fdxCN* genotype) abolished diazotrophic growth via the Fe‐nitrogenase in *R. capsulatus*, while the removal of either *fdxC* or *fdxN* (∆*fdxC* and ∆*fdxN* genotypes) resulted in a roughly three‐to‐four‐fold increase in doubling time (*t*_d_) compared to the wild‐type strain (WT; *t*_d_ = ∼11 hours; Table 1). Specifically, the ∆*fdxC* strain has a *t*_d_ of ∼44 hours, while the ∆*fdxN* strain has a *t*_d_ of *∼*29 hours (Table 1). The slow growing deletion strains also showed a decreased in vivo Fe‐nitrogenase activity, the ∆*fdxN* strain shows an activity of 25%, and ∆*fdxC* strain an activity of 10% compared to WT.^[^ ^17^ ^]^ It remains unclear why the loss of either FdC or FdN hampers N_2_‐fixation, despite the expected differences in their properties. Hence, we first explored if FdC and FdN are adapted for different roles or if they are interchangeable with each other within the cell. To investigate this, ∆*fdxC* and ∆*fdxN R. capsulatus* deletion strains were complemented individually by single‐copy number plasmids encoding either *fdxC* and *fdxN* (Figure 2; Table 1).^[^ ^28^ ^]^

**Table 1:** Doubling times of *fdx* complemented *R. capsulatus* strains.

Genotype	Doubling time [*t*_d_] [h]
WT	11.1 ± 0.8
∆*fdxC*	43.7 ± 21.2^[^ ^17^ ^]^
*∆fdxN*	28.7 ± 4.4^[^ ^17^ ^]^
∆*fdxC fdxC*	17.1 ± 1.3
∆*fdxC fdxN*	17.9 ± 1.6
∆*fdxN fdxN*	11.3 ± 0.5
∆*fdxN fdxC*	29.3 ± 0.6
∆*anfDGK*	>100

An exponential Malthusian growth model was used to determine individual doubling times (*t*_d_). Only OD_660_ values in the linear range were included. The linear range was defined as values between 0 hour and 43.5 hours for strains WT, ∆*fdxN fdxN*, ∆*anfDGK*. For strains ∆*fdxC fdxC*, ∆*fdxC fdxN*, and ∆*fdxN fdxC* the linear range was from 0 hours until 70.5 hours. The means and standard deviation of the *t*_d_ values were calculated using three biological replicates.

Surprisingly, introducing a plasmid‐encoded copy of either *fdxC* or *fdxN* in the ∆*fdxC* strain recovered both the diazotrophic growth and Fe‐nitrogenase activity (Figure 2A,B). Diazotrophic growth recovery was similar for both ∆*fdxC fdxC* and ∆*fdxC fdxN*, highlighted by the comparable *t*_d_ values of 17–18 hours of both strains, compared to the ∆*fdxC* parent strain which has a *t*_d_ of ∼44 hours (Table 1).^[^ ^17^ ^]^ This similar growth behavior of ∆*fdxC fdxC* and ∆*fdxC fdxN* was corroborated by statistically indistinguishable in vivo Fe‐nitrogenase activities, for both ethylene and ethane formation from acetylene, by the two strains (Figure 2B). The inability of *fdxC* expression to fully complement ∆*fdxC* has been reported prior and may be caused by a mismatch of the protein level of FdC (either slightly lower or higher) in the complementation strain relative to the WT strain, therefore negatively impacting cellular redox homeostasis.^[^ ^17^ ^]^

The interchangeability of *fdxC* and *fdxN* was interesting, as it appeared that *fdxN* was able to recover ∆*fdxC* phenotypes to the same levels as *fdxC* encoded by a plasmid. Indeed we had observed previously that FdN is one of the most highly upregulated proteins in the ∆*fdxC* strain compared to the WT strain, identified by whole cell proteomics, suggesting an important role of FdN in N_2_‐fixation when FdC is removed.^[^ ^17^ ^]^ However, the result is surprising, since FdN is expected to contain two [Fe_4_S_4_]‐clusters while FdC is expected to contain only one [Fe_2_S_2_]‐cluster, also implying large differences in redox potentials.^[^ ^18^, ^19^, ^24^, ^29^ ^]^ One hypothesis for this exchangeability of FdC with FdN is that the highly reducing nature of FdN allows it to substitute the function of the less reducing FdC, with a ∼200 mV higher redox potential (see below). It could be that FdN and FdC interact with partner proteins using unspecific charged patches, rather than specific residue interactions, as shown previously for Fd interactions with cysteine desulfurase,^[^ ^30^ ^]^ and this might allow interchangeability of FdC and FdN.^[^ ^30^ ^]^ It has been shown in other cases that small electron carrier proteins can be interchanged for each other, specifically in *R. palustris* it was shown that Fld becomes a prominent electron donor to the Mo‐nitrogenase, over low‐potential Fds, in Fe‐deplete growth conditions.^[^ ^31^ ^]^

On the contrary, only introduction of a plasmid‐encoded copy of *fdxN*, not an additional copy of *fdxC*, into the ∆*fdxN* strain recovered diazotrophic growth and Fe‐nitrogenase activity (Figure 2C and D). When *fdxC* was introduced into the ∆*fdxN* strain, there was no recovery of the slower growth phenotype of the ∆*fdxN* strain, with both strains having *t*_d_ values of ∼29 hours, (Figure 2C, Table 1).^[^ ^17^ ^]^ Conversely, the ∆*fdxN fdxN* complementation strain has a *t*_d_ of ∼11 hours, equivalent to the *t*_d_ of the WT strain (Figure 2C, Table 1). As expected, the Fe‐nitrogenase activity of the ∆*fdxN fdxC* strain was lower than the WT, around 25% for both ethylene and ethane evolution (Figure 2D). The ∆*fdxN fdxN* strain had similar activity to the WT, and significantly higher Fe‐nitrogenase activity than the ∆*fdxN fdxC* strain (Figure 2D). The results showed that *fdxC* expression could not complement the loss of *fdxN*. This observation was likely because the reduction potential of FdC is too positive to transfer electrons to the reductase component (AnfH_2_) of the Fe‐nitrogenase, the suggested function of FdN.^[^ ^32^ ^]^

### 2.2 FdC Is Monomeric and Coordinates a [Fe_2_S_2_]‐cluster of Potential –285 mV

To understand the different complementation behaviors of FdC and FdN in vivo, the next step was to purify, spectroscopically and structurally characterize FdC and FdN in vitro, to define the properties that cause both of these Fds to be in combination essential for N_2_‐fixation.

**Figure 3:**
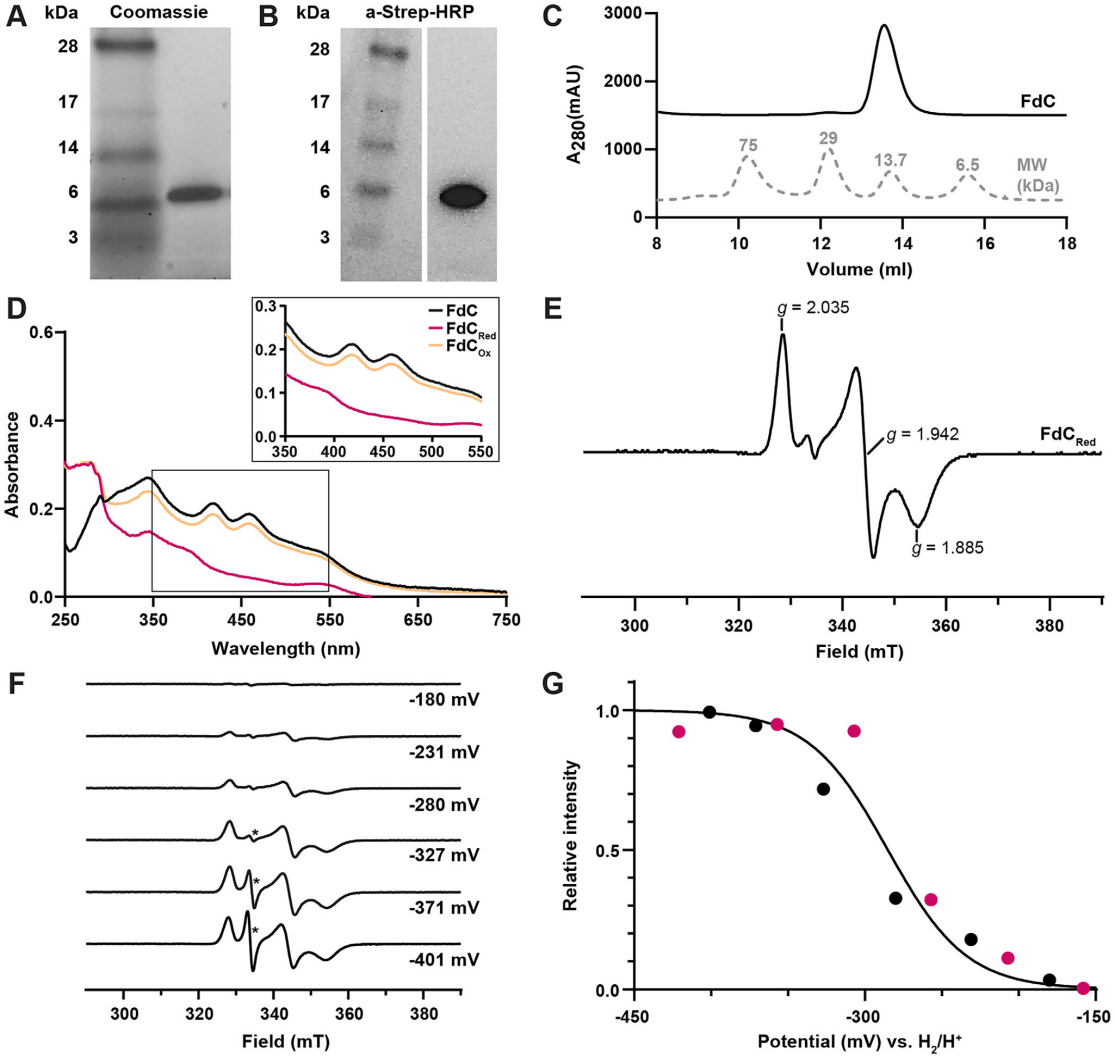
Purification and spectroscopic characterization of *R. capsulatus* ferredoxin C. **(A, B)** Protein electrophoresis of FdC stained with **(A)** Coomassie for SDS‐Page analysis and **(B)** *a*‐Strep‐HRP for western blot analysis. Ladder is SeeBlue Plus2 prestained ladder. **(C)** Size exclusion chromatography trace of FdC with low MW standards shown. **(D)** UV‐Vis absorbance spectra of FdC in the wavelength range of 250 nm–750 nm. FdC traces are in the absence of reductant (FdC, black), reduced with sodium dithionite (FdC_red_, pink) and oxidized by exposure to air (FdC_ox_, yellow). Highlighted in the box is the wavelength range (350 nm – 550 nm) where unique features of the [Fe_2_S_2_]‐cluster containing FdC are observed. **(E‐G)** EPR spectroscopy of FdC at 15 K. EPR conditions: microwave frequency 9.35 GHz, 13 µW microwave power, 1.0 mT modulation amplitude (0.4 mT in panel E). **(E)** EPR spectrum of the *S* = ½ region of the reduced FdC at pH 7.8, with *g* values indicated. **(F)** EPR spectra of a reductive titration of FdC. Samples are poised at the reduction potentials indicated, between ‐401 mV and ‐180 mV. The pronounced signal at *g *= 2.002 (*) is from semiquinone radicals of the methyl‐ and benzylviologen mediators. **(G)** Normalized EPR amplitudes of FdC spectra from two independent reductive titrations, fit to the Nernst equation with n = 1 and an E_m_ of–285 mV. Points represent individual samples and are either black, for the titration shown in **(F)**, or pink, for a second separate titration.

FdC was heterologously produced in and purified from *Escherichia coli*, a commonly used prokaryotic host for recombinant protein production. *Escherichia coli* was grown under micro‐aerobic conditions while all subsequent protein purification steps were performed anaerobically. Upon streptactin (strep) affinity purification of FdC an elution fraction with a strong red color was obtained, indicating the presence of a [Fe_2_S_2_]‐cluster (Figure S1).^[^ ^33^ ^]^ Analysis by sodium dodecylsulfate polyacrylamide gel electrophoresis (SDS‐Page) revealed a single band corresponding to the strep‐tagged FdC with an apparent molecular weight (MW) of just over 6 kDa (Figure 3A). As the MW of the FdC band differed from the calculated MW of FdC (11.3 kDa), we confirmed that the band at 6 kDa was strep‐tagged FdC via western blot (WB) transfer of the protein after SDS‐Page and detection of the strep‐tag with anti‐strep monoclonal antibodies linked to peroxidase (Figure 3B, Table S2).

To investigate the oligomeric state of purified FdC, size exclusion chromatography (SEC) was performed (Figure 3C). FdC eluted from the column as a single homogenous peak at a determined MW of 16 kDa, demonstrating that FdC is a monomer.

As FeS‐cluster proteins are known to produce distinct spectral features in the absorption range of 300 nm–750 nm, UV‐Vis spectra for FdC were collected (Figure 3D). The absorbance features of FeS‐cluster containing proteins in this range are dependent on both the type and redox state of the FeS‐cluster they contain. The measured UV‐Vis spectra with two characteristic peaks, at 418 nm and 458 nm establishes the presence of a [Fe_2_S_2_] ^2+^‐cluster in the purified FdC.^[^ ^34^ ^]^ These features in the FdC spectra were very similar to other spectra of bacterial [Fe_2_S_2_]‐cluster containing Fds from *Aquifex aeolicus* and *Acidithiobacillus ferrooxidans*.^[^ ^35^, ^36^ ^]^ Upon reduction of FdC, the two peaks disappeared indicating the reduction of the [Fe_2_S_2_]‐cluster to the [Fe_2_S_2_]^1+^ form. When FdC was re‐oxidized by treatment with air the spectrum shows again the characteristic peaks of the [Fe_2_S_2_]^2+^ form, but with a 10% loss of chromophore. The UV‐Vis spectra under oxidizing and reducing conditions demonstrate the presence of a functional and redox active [Fe_2_S_2_]‐cluster.

The electronic structure of the [Fe_2_S_2_]‐cluster within FdC was further analyzed by EPR spectroscopy (Figure 3E‐G).^[^ ^34^ ^]^ Unique spectra are produced by the presence of unpaired electrons in FeS‐clusters, that is, in the [Fe_2_S_2_]^1+^ and [Fe_4_S_4_]^1+^ forms. By the addition of sodium dithionite, a reduced sample of FdC was prepared, producing a characteristic signal in the *S* = ½ region that was rhombic in shape with associated *g*‐values of *g* = 2.035, 1.942, and 1.885 (Figure 3E). The rhombic signal (*g*_average _= 1.954) is typical for the [Fe_2_S_2_]‐cluster of the adrenodoxin type (*g *= 1.961 ± 0.005, *n* = 78) in the single‐electron reduced form ([Fe_2_S_2_]^1+^).^[^ ^37^ ^]^

To determine the reduction potential of FdC, reductive redox titrations were performed between potentials of −150 mV and −420 mV (Figure 3F,G). Combination of the relative intensities of the derivative shaped *g *= 1.942 EPR signal (Figure 3F) from two separate redox titrations allowed for the determination of a midpoint potential of −285 ± 10 mV for FdC (Figure 3G). The [Fe_4_S_4_]^1+/2+^ cluster of nitrogenase reductase components typically has a redox potential in the presence of cellular concentrations of ATP or ADP (mM) below ‐400 mV.^[^ ^38^ ^]^ Currently, there is no reported reduction potential for AnfH_2_, though it is likely in a similar range to the reductase component of the homologous Mo‐nitrogenase (NifH_2_).^[^ ^14^, ^39^ ^]^ To summarize, the spectroscopic characterization of monomeric FdC confirms the presence of a redox active [Fe_2_S_2_]‐cluster with a midpoint reduction potential of −285 ± 10 mV, which is unlikely to be an efficient electron donor to the reductase component of nitrogenase, including the *R. capsulatus* Fe‐nitrogenase system.

### 2.3 FdN Coordinates Two [Fe_4_S_4_]‐clusters and Has Unique EPR Spectra

Unlike for FdC, we were not able to purify a fully occupied FdN holoprotein from *E. coli*. Only purification from the native host *R. capsulatus* under strictly anaerobic conditions yielded FeS‐cluster replete FdN. This was likely due to the lability and high‐oxygen sensitivity of the two predicted [Fe_4_S_4_]‐clusters in FdN.

**Figure 4:**
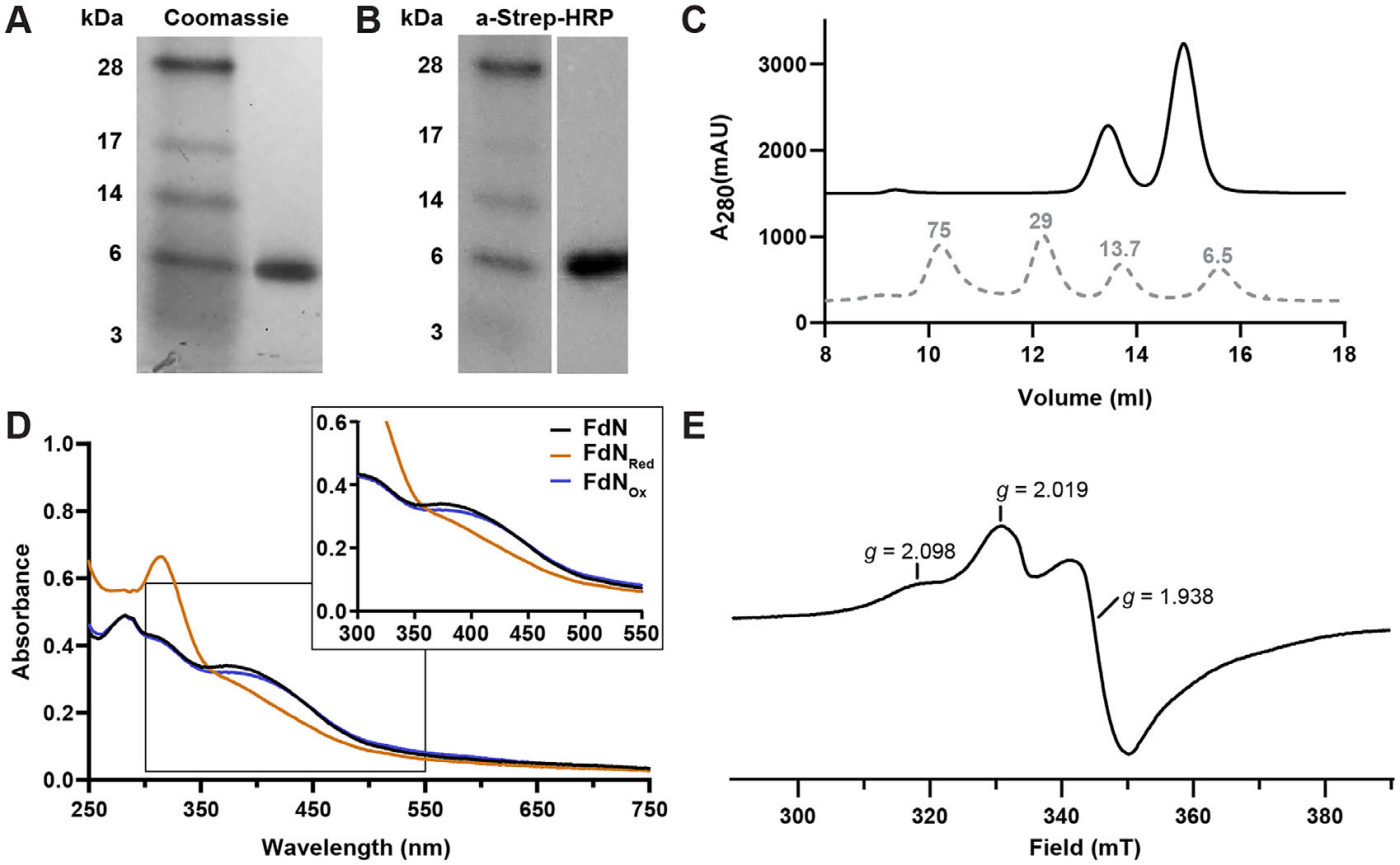
Purification and spectroscopic characterization of *R. capsulatus* ferredoxin N. **(A, B)** Protein electrophoresis of FdN stained with **(A)** Coomassie for SDS‐Page analysis and **(B)** *a*‐Strep‐HRP for western blot analysis. Ladder is SeeBlue Plus2 prestained ladder. **(C)** Size exclusion chromatography trace of FdN with low MW standards shown (same low MW equilibration mix as shown in Figure 3C). **(D)** UV‐Vis spectra of FdN. FdN traces are in the absence of reductant (FdN, black), reduced with sodium dithionite (FdN_red_, brown) and oxidized by exposure to air (FdN_ox_, blue). Highlighted in the box is the wavelength range (300 nm–550 nm) showing the unique features of the two [Fe_4_S_4_]‐clusters within FdN. **(E)** EPR spectrum at 10 K of the *S* = ½ region of the reduced FdN at pH 9.0, with *g* values indicated. EPR conditions: microwave frequency 9.35 GHz, 0.2 mW microwave power, 1.0 mT modulation amplitude.

Strep‐purification of FdN produced a dark brown elution fraction, characteristic of [Fe_4_S_4_]‐cluster containing proteins (Figure S1). The FdN elution fraction was analyzed via SDS‐Page gel electrophoresis, where the prominent band corresponding to FdN appeared at around 6 kDa (Figure 4A). The mobility is in moderate agreement with the calculated MW of C‐terminally strep‐tagged FdN (7.9 kDa, Table S2). Again, we confirmed that the band was from strep‐tagged FdN using anti‐strep western blot analysis (Figure 4B).

Running the FdN elution fraction on a SEC column produced two peaks at MWs of around 8.5 kDa and 16.5 kDa, likely corresponding to a monomeric FdN and a dimeric FdN form, respectively (Figure 4C). The larger peak was the monomeric peak at 8.5 kDa, indicating the physiologically predominant species may be the monomeric form. The FdN dimer may have been an artefact, only occurring due to unphysiologically high concentrations of FdN, > 10 mg/mL.

To identify the contained FeS‐clusters, UV‐Vis absorption spectra of FdN was collected in the range of 250 –750 nm (Figure 4D). The UV‐Vis spectra of FdN are characteristic of [Fe_4_S_4_]‐cluster containing proteins, producing a broad peak in the wavelength range of 350–500 nm. This broad peak represents the oxidized [Fe_4_S_4_]^2+^‐clusters of FdN. Upon reduction of FdN, the peak only partially bleached indicating incomplete reduction of the clusters to their [Fe_4_S_4_]^1+^ form. Normally approximately 50% bleaching is observed upon complete reduction.^[^ ^40^ ^]^ When FdN was re‐oxidized, the broad peak in 350–500 nm range almost completely reappeared; representing the clusters in the [Fe_4_S_4_]^2+^ form. Functional assembly of the FdN [Fe_4_S_4_]‐clusters was demonstrated by the successful chemical reduction and re‐oxidation of FdN.

To investigate the electronic structure of the two [Fe_4_S_4_]‐clusters within FdN, EPR spectra were collected (Figure 4E). Reduction of FdN by the addition of sodium dithionite was only successful at pH values above 8 (i.e., below ‐500 mV). ^[^ ^41^ ^]^ At pH 9 the sample producing a complex EPR signal in the *S* = ½ region, which was not a sum of axial or rhombic species, but from its width indicated spin‐spin interaction between the two [Fe_4_S_4_]‐clusters.^[^ ^42^ ^]^ The behavior of FdN differed from other two [Fe_4_S_4_]‐cluster Fds. For example, the two [Fe_4_S_4_]‐cluster *C. pasteurianum* Fd shows a clear spectral change from the single to the double (2 x [Fe_4_S_4_]^1+^) reduced state, upon treatment with sodium dithionite.^[^ ^43^ ^]^ For FdN, the changes seem to indicate redox cooperativity: the reduction of both clusters is favoured in comparison with sequential reduction. These findings match a prior study, in which the reduction potentials of both [Fe_4_S_4_]‐clusters within FdN were determined to be −490 mV.^[^ ^18^ ^]^ These reduction potentials mean that FdN is a strong reducing agent able to donate electrons to the reductase component of the Fe‐nitrogenase. Importantly, FdN is almost 200 mV more reducing than FdC with a reduction potential of −285 ± 10 mV. This difference in the reduction potentials between FdN and FdC supports our hypothesis that the low reduction potential of FdN allows it to replace FdC as an electron donor in vivo. In conclusion, FdN contains two [Fe_4_S_4_]‐clusters with a low reduction potential as revealed by UV/Vis and EPR spectroscopy.

### 2.4 FdC Crystal Structure Reveals a Novel Tertiary Structure around the [Fe_2_S_2_]‐cluster

Next, we used X‐ray crystallography of FdC and structural modeling of FdN to study the molecular architectures of FdC and FdN. The aims of the structural investigations were to uncover the secondary structure of both Fds, define the coordination of their active site cofactors and to provide insights into the surface residues potentially mediating interactions with partner proteins.

**Figure 5:**
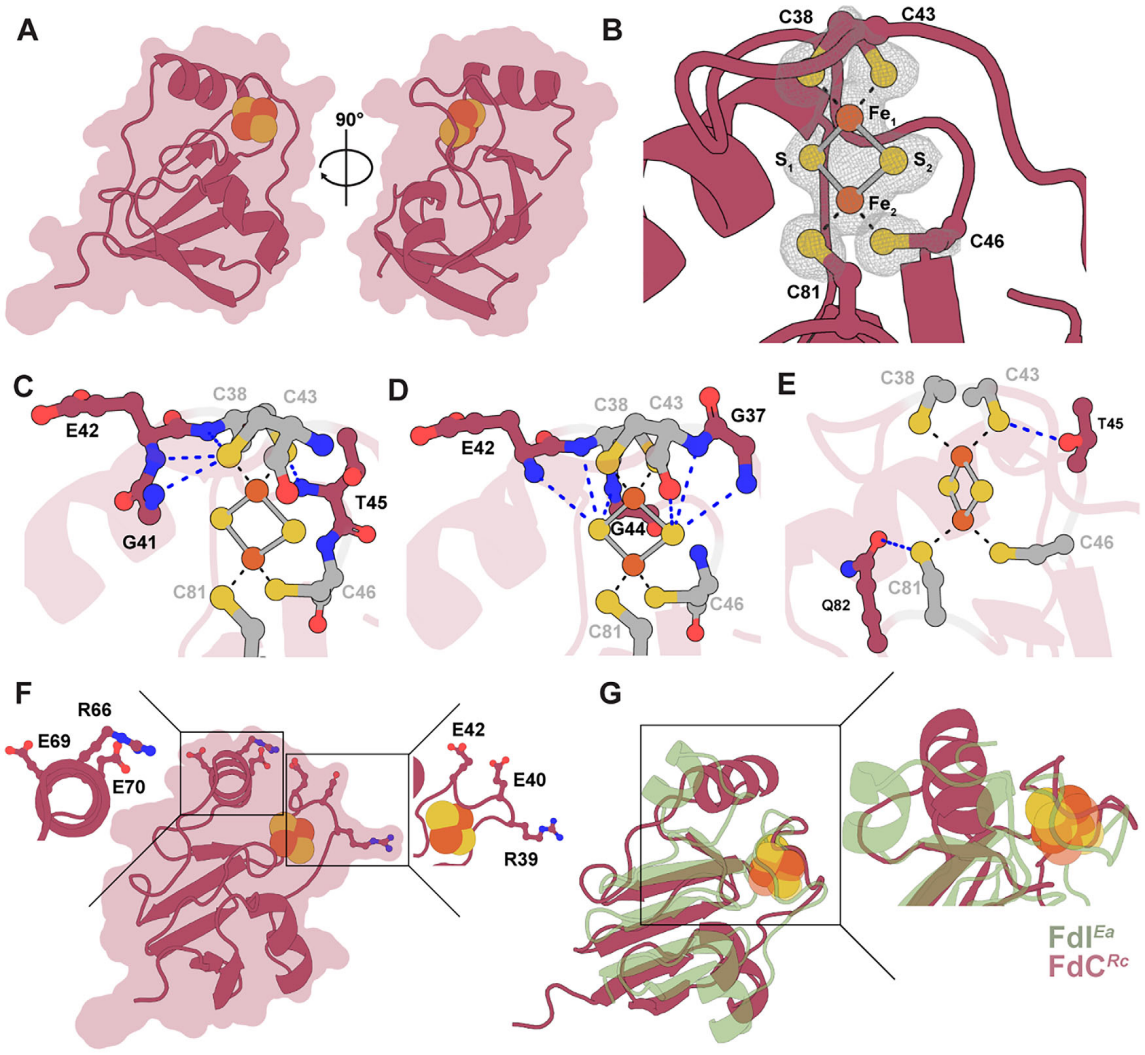
The FdC structure reveals the environment of the [Fe_2_S_2_]‐cluster. **(A)** Protein structure of the FdC monomer shown in an overlaid cartoon and surface representation. The model is rotated by ‐90° in the y‐axis plane to show another view. **(B)** Omit map (black mesh, σ = 3.0) of [Fe_2_S_2_]‐cluster and coordinating Cys residues (dashed black lines): C38, C43, C46, and C81. **(C‐E)** Residues contributing to the hydrogen‐bonding network around the [Fe_2_S_2_]‐centre in FdC. Coordinating Cys residues are shown in grey. Blue dashed lines indicate hydrogen bonds. **(C)** Residues form hydrogen‐bonds with the thiolate side chains of coordinating Cys residues. **(D)** Residues form hydrogen‐bonds with the sulfides of the [Fe_2_S_2_]‐cluster. **(E)** Hydrogen‐bonds formed between the side chains of residues T45 and Q82 and the [Fe_2_S_2_]‐cluster. **(F)** Magnification of charged residues in close proximity to the [Fe_2_S_2_]‐cluster of FdC. (Left box) Charged residues situated on the top helix. (Right box) Charged residues situated on the loop. **(G)** (Left) Comparison of FdC (shown in red) and ferredoxin I from *Equisetum arvense* (PDB: 1FRR) (shown in green), with a magnification on the regions with the [Fe_2_S_2_]‐clusters (right). **(A‐G)** FeS‐clusters and highlighted residues are shown as ball‐and‐stick models with iron in orange, sulfur in yellow, oxygen in red and nitrogen in blue.

A 1.7 Å final resolution model of FdC and the coordination of the [Fe_2_S_2_]‐cluster was constructed (PDB 9I2A) using X‐ray crystallography (Figure 5). The globular and monomeric FdC coordinates a [Fe_2_S_2_]‐cluster (Figure 5A, Figure S3). This [Fe_2_S_2_]‐cluster is located within 5 Å of the protein surface, likely to facilitate rapid electron transfer to partner proteins.

FdC was found to comprise of five β‐sheets and two α‐helices, one flanking the β‐sheets and one situated on top of the protein close to the [Fe_2_S_2_]‐cluster. The β‐sheets and the flanking α‐helix together are a specific feature called the β‐grasp fold, which is observed in a diverse range of functionally unrelated protein families.^[^ ^44^ ^]^ Notably, the β‐grasp fold is frequently found in other [Fe_2_S_2_]‐cluster containing Fds, that is, in plant‐type Fds from *Spirulina platensis*,^[^ ^45^ ^]^ *Anabaena* ^[^ ^46^ ^]^ and *E. arvense*.^[^ ^47^ ^]^ The β‐grasp fold provides mechanical stability within proteins; it has been shown that the Fd from *Anabaena* couples the stabilizing effect of the β‐grasp fold with its [Fe_2_S_2_]‐cluster to achieve further protein stabilization.^[^ ^48^ ^]^

The [Fe_2_S_2_]‐cluster of FdC was coordinated by the thiolate side chains of four Cys residues (C38, C43, C46, and C81) in a planar fashion (Figure 5B). The bond lengths of each coordinate bond between the Fe ions of the [Fe_2_S_2_]‐cluster and thiolate ions of the Cys residues were very similar, with lengths of ∼2.3 Å (Table S4). There were also equivalent bond lengths between each Fe ion and each inorganic S^2−^ ion, with distances of ∼2.2 Å (Table S4). These bond lengths within the [Fe_2_S_2_]‐cluster of FdC were very similar to crystallographically determined distances of [Fe_2_S_2_]‐cluster containing Fds from cyanobacteria and plant species.^[^ ^49^ ^]^ Notably, a fifth Cys residue, C83, appeared in the centre of FdC, though it does not form coordinate bonds with the [Fe_2_S_2_]‐cluster.

Since hydrogen bonding networks around the FeS‐clusters within Fds are important for function and stabilizing the active site, we closely scrutinized the extensive hydrogen bonding network of FdC.^[^ ^47^ ^]^ Overall, we identified 13 interactions, around the [Fe_2_S_2_]‐cluster (Figure 5C‐E, Table 2). These hydrogen bonds are formed between partially positively charged hydrogen atoms (H^δ+^) and either the negatively charged S^2−^ ions of the FeS‐cluster itself or the thiolate (S^−^) side chains of the coordinating Cys residues. Only two polar residues (threonine 45 and glutamine 82) had side chains in close proximity to form hydrogen bonds with either the S^2−^ ions or the thiolate side chains. Notably, this differed from structures of other plant and algal‐type Fds which rely on hydrogen bonds created with either one or two serine side chains to stabilize the active site.^[^ ^45^, ^47^, ^50^, ^51^ ^]^

**Table 2:** Hydrogen‐bonding network around the [Fe_2_S_2_] centre in FdC^[a]^

Bond	Distance [Å]	Bond	Angle [°]	Bond	Angle [°]
38S^γ^‐41N	3.2	41N‐38S^γ^‐38C^β^	140.0	41N‐38S^γ^‐Fe_1_	108.0
38S^γ^‐42N	3.4	42N‐38S^γ^‐38C^β^	134.6	42N‐38S^γ^‐Fe_1_	91.9
38S^γ^‐43N	3.6	43N‐38S^γ^‐38C^β^	103.0	43N‐38S^γ^‐Fe_1_	69.9
43S^γ^‐45N	3.4	45N‐43S^γ^‐43C^β^	104.8	45N‐43S^γ^‐Fe_1_	105.7
43S^γ^‐45O^β^	3.1	45O^β^‐43S^γ^‐43C^β^	126.7	45O^β^‐43S^γ^‐Fe_1_	112.7
81S^γ^‐82O^δ^	3.8	82O^δ^‐81S^γ^‐81C^β^	150.1	82O^δ^‐81S^γ^‐Fe_2_	96.7
S_1_‐42N	3.7	42N‐S_1_‐Fe_2_	135.6	42N‐S_1_‐Fe_1_	85.9
S_1_‐43N	3.6	43N‐S_1_‐Fe_2_	145.6	43N‐S_1_‐Fe_1_	70.1
S_1_‐44N	3.1	44N‐S_1_‐Fe_2_	126.5	44N‐S_1_‐Fe_1_	86.2
S_2_‐37N	3.5	37N‐S_2_‐Fe_1_	102.9	37N‐S_2_‐Fe_2_	158.2
S_2_‐38N	3.7	38N‐S_2_‐Fe_2_	147.4	38N‐S_2_‐Fe_1_	74.2
S_2_‐38O	3.6	38O‐S_2_‐Fe_2_	125.5	38O‐S_2_‐Fe_1_	87.6

^[a]^Numbers in the columns for the bonds correspond to the position of the amino acid in the primary sequence, N is for the nitrogen atom of the amino group, O for the oxygen atom of the carbonyl of the peptide bonds. S1, S2, Fe1, and Fe2 for the [Fe_2_S_2_]‐cluster. Other atoms are the side chains of the denoted amino acids. Distances are shown correct to one decimal place. Bond distances and angles were calculated using PyMOL.^[^ ^52^ ^]^

Residues in close proximity to the [Fe_2_S_2_]‐cluster of FdC were analyzed to identify potential residues that could be involved in binding partner proteins or mediating electron transfer to these partners (Figure 5F). We discovered that FdC has many outward facing charged residues in close proximity to the [Fe_2_S_2_]‐cluster. There were two interesting triads of residues on both the top helix of FdC and on the [Fe_2_S_2_]‐cluster coordinating loop region. On the top helix, there were two negatively charged outward facing glutamic acid (Glu) residues (E69 and E70) and one positively charged outward facing arginine (Arg) residue (R66). Additionally, very close to the [Fe_2_S_2_]‐cluster in the loop region, there were two negatively charged Glu residues (E40 and E42) and a negatively charged Arg residue (R39). Both triads could be involved in forming salt bridges between FdC and its partner proteins. Moreover, the surface region surrounding the [Fe_2_S_2_]‐cluster has a largely negative charge perhaps important for mediating electrostatic interactions with partner proteins, as observed with other Fds and their partners (Figure S4).^[^ ^30^, ^50^ ^]^ In other studies it has also been shown that Fds interact with their partners via specific surface residues close to their active site clusters.^[^ ^50^ ^]^ Specifically, key surface exposed residues, including but not exclusive to aspartic acid (Asp) and Glu residues, on both *E. coli* FdI and *Homo sapiens* FDX2 enable interaction with cysteine desulfurase proteins or complexes.^[^ ^30^, ^53^ ^]^ Recently, this has been corroborated by a cryogenic electron microscopy structure of the mitochondrial core FeS‐cluster assembly (ISC) complex. Here, it was shown that *H. sapiens* FDX2 formed salt bridges with cysteine desulfurase, which were created with negatively charged Glu and Asp residues on FDX2 in close proximity to the [Fe_2_S_2_]‐cluster.^[^ ^30^ ^]^

To assess how the FdC structure differs from other structurally characterized Fds, FdC was compared to the most similar Fd structure in the Protein Data Bank (PDB), which was FdI (FdI*^Ea^*) from the herbaceous plant species *E. arvense*.^[^ ^47^ ^]^ To compare FdC with FdI*^Ea^*, an overlay of the two structures was created (Figure 5G). The overlaid structures had a calculated global root mean square deviation (RMSD) between backbone carbons (Cα) of 4.86 Å (68 to 68 atoms), highlighting structural differences between the two Fds. This variation was mainly due to differences in the helices and loop regions located near the [Fe_2_S_2_]‐cluster. The β‐grasp regions, which both Fds use to maintain stability, had similar arrangements in both Fd structures. Considering the two Fds originate from organisms with very different metabolisms and ecological niches, these findings suggest that the structural differences probably originate from the formation of specific interactions with different partner proteins. In summary, the structure of FdC revealed the coordination of a [Fe_2_S_2_]‐cluster with a unique tertiary structure surrounding this cluster compared to other similar solved structures of [Fe_2_S_2_]‐cluster Fds.

### 2.5 AlphaFold Model of FdN Reveals an Extra Loop in Close Proximity to One [Fe_4_S_4_]‐cluster

**Figure 6:**
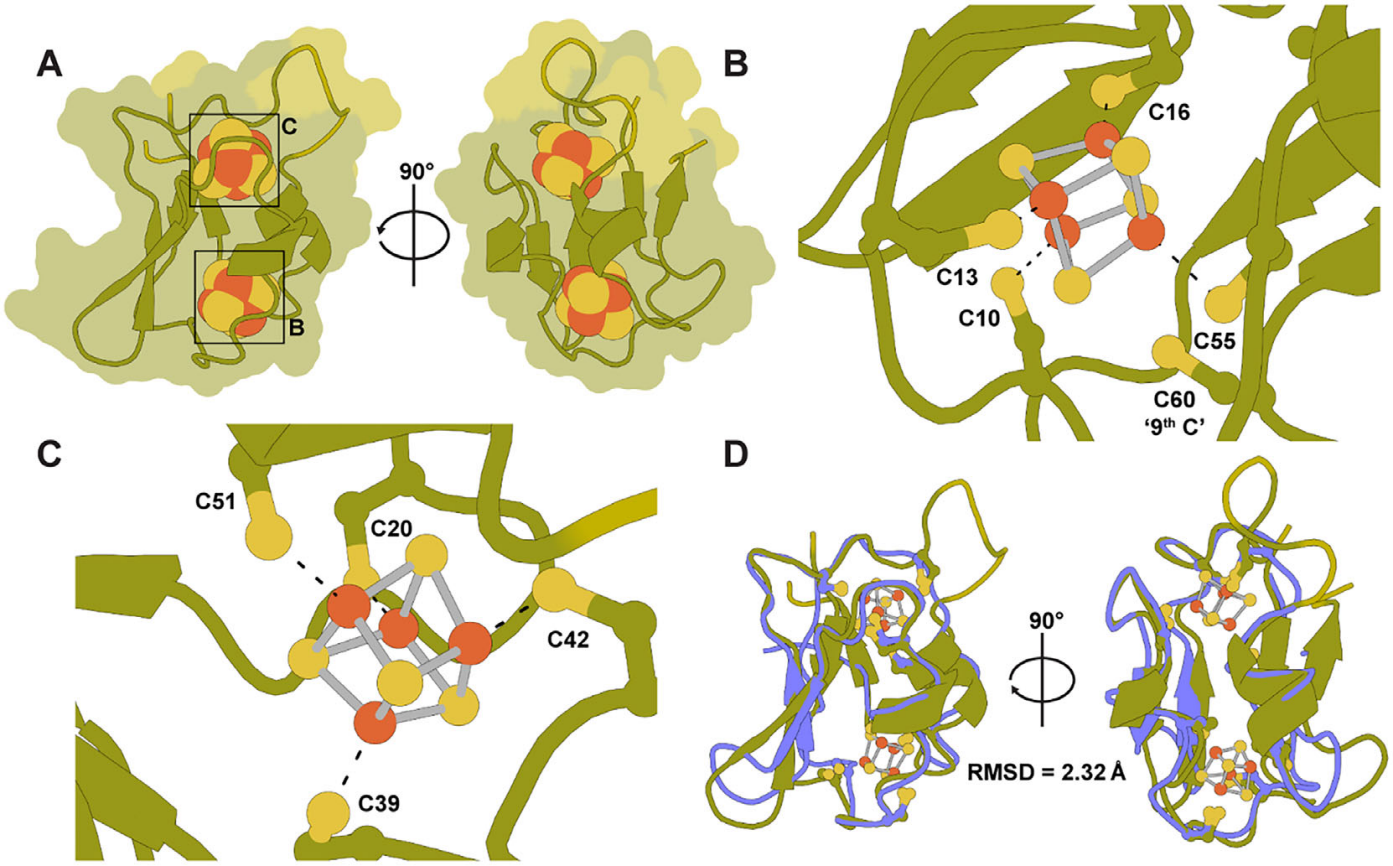
AlphaFold model of FdN containing two [Fe_4_S_4_]‐clusters. **(A‐D)** AlphaFold model of FdN (AF‐D5ARY6), overlaid with [Fe_4_S_4_]‐clusters from *C. pasteurianum* ferredoxin (PDB: 1CLF).^[^ ^6^ ^]^ **(A)** FdN model shown in an overlaid cartoon and surface representation. 90% of residues in the FdN AlphaFold model have very high predicted local distance difference test (pLDDT > 90) values shown in deep olive, while 10% of the residues have high confidence values (90 > pLLDT > 70) shown in olive. The two [Fe_4_S_4_]‐clusters are magnified in panel B and C as labelled. **(B‐C)** [Fe_4_S_4_]‐clusters and coordinating cysteine residues of FdN are shown in ball‐and‐stick representation. Black dashed lines represent coordinate bonds between Cys residues and Fe ions. **(D)** Overlay of FdN AlphaFold model (AF‐D5ARY6) and *C. pasteurianum* ferredoxin (1CLF) in cartoon representation. 1CLF is colored blue (color slate). [Fe_4_S_4_]‐clusters of 1CLF and coordinating cysteine residues of both Fds are shown in stick representation. The global RMSD between the backbone carbons (Cα) of the two proteins is 2.32 Å as indicated.

Lacking experimental structural data, we used a high confidence AlphaFold model to investigate the structural features of FdN, visualized with two [Fe_4_S_4_]‐clusters modelled‐in from the structure of *C. pasteurianum* Fd (PDB: 1CLF) (Figure 6A).^[^ ^6^, ^54^, ^55^ ^]^ FdN also closely resembles the structures of other two [Fe_4_S_4_]‐cluster Fds (PDB: 1DUR, 2FDN).^[^ ^56^ ^]^ The Alphafold FdN model had nine conserved Cys residues, the first eight coordinating the two [Fe_4_S_4_]‐clusters in a typical arrangement for cubane FeS‐cluster coordination (Figure 6B,C). The ninth Cys residue, C60, is a nonligating Cys residue, situated in close proximity to one of the [Fe_4_S_4_]‐clusters (“cluster B” shown in Figure 6B). C60 does not coordinate either of the [Fe_4_S_4_]‐clusters in FdN.^[^ ^18^ ^]^ In general, the 9^th^ Cys residues have instead been suggested to tune the redox potentials of [Fe_4_S_4_]‐clusters within several Fds. This is achieved by the formation of hydrogen bonds between the thiol side chain of the nonligating Cys residue and the sulfide ions present in the cluster.^[^ ^57^ ^]^ The extra hydrogen bond creates a stabilizing effect on the [Fe_4_S_4_]‐cluster, which is accompanied by a shift in the protein backbone away from the redox centre, which leads to an overall lowering of the reduction potential.

Since *R. capsulatus* FdN has a quite different primary structure compared with well‐characterized two [Fe_4_S_4_] Fds from nondiazotrophic organisms, the FdN AlphaFold model was compared with *C. pasteurianum* Fd (Figure 6D).^[^ ^6^ ^]^ Overall, the Fd protein structures were very similar with a RMSD value of only 2.32 Å (47 of 47 atoms). However, a key difference between the structures of *R. capsulatus* FdN and *C. pasteurianum* Fd was an additional loop region around one of the two [Fe_4_S_4_]‐clusters in *R. capsulatus* FdN. This difference between the two structures arose from differences in the arrangement of the first eight conserved Cys residues. In *C. pasteurianum* Fd, the conserved sequence describing the coordinating Cys residues for the two [Fe_4_S_4_]‐clusters is comprised of a tandem arrangement of primary structural elements.^[^ ^58^ ^]^ This motif typically is Cys‐X_2_‐Cys‐X_2_‐Cys‐X_3_‐Cys‐Pro, where X denotes any amino acid except Cys, and the first three Cys residues coordinate one [Fe_4_S_4_]‐cluster while the last Cys coordinates the other [Fe_4_S_4_]‐cluster and vice versa.^[^ ^59^ ^]^ On the other hand, *R. capsulatus* FdN has one Cys‐X_2_‐Cys‐X_2_‐Cys‐X_3_‐Cys sequence but the second part of the motif has an additional six residues between Cys residues two and three: Cys‐X_2_‐Cys‐X_8_‐Cys‐X_3_‐Cys.^[^ ^18^ ^]^ These additional residues make up the extra loop region around one of the [Fe_4_S_4_]‐clusters. Further, the last Cys residue in the second motif of FdN does not have a vicinal proline residue at the C‐terminal end, unlike *C. pasteurianum* Fd.

It is unknown why exactly there is an extra loop in *R. capsulatus* FdN compared with *C. pasteurianum* Fd, as previously it was shown that the replacement of the extra loop residues 42–49 residues by Gly‐Ala did not impact the ability of *R. capsulatus* to fix N_2_.^[^ ^18^ ^]^ It is surprising that the extension of the loop is conserved in several other known electron donors to nitrogenase, specifically Fer1 and FerN of *R. palustris* and FdxN of *Sinorhizobium meliloti*.^[^ ^31^, ^60^, ^61^ ^]^ Overall, the model of FdN revealed a nonsymmetrical structure with the tertiary structure around one of the two [Fe_4_S_4_]‐clusters closing resembling *C. pasteurianum* Fd, while the tertiary structure around the other [Fe_4_S_4_]‐cluster had a different conformation, notably an additional loop region. These structural differences may be the cause of the observed redox properties FdN and *C. pasteurianum* Fd.

## 3 Discussion

Here, we systematically characterized FdC and FdN, which are collectively essential for N_2_‐fixation by the Fe‐nitrogenase in *R. capsulatus*. Overall, our work provides new insights into the open question as to why many organisms possess multiple Fds despite their similar functions as electron donors and acceptors.

We demonstrate through in vivo and in vitro experiments that both proteins differ in physiological function, interaction specificity with partner proteins, structure, and electronic properties. Using complementation studies, we show that FdN and FdC take different physiological roles. We found that there is a degree of interchangeability between FdN and FdC in vivo, though this is asymmetrically, meaning *fdxN* can complement both a ∆*fdxC* and a ∆*fdxN* strain while *fdxC* can only complement the ∆*fdxC* strain. This surprising result showed some level of functional redundancy between both Fds and suggested there are features of FdN that allow it to recover the ∆*fdxC* phenotype. Our hypothesis is that FdN has a much lower reduction potential than FdC and thus it can substitute for FdC, performing its role in the cell, at least indirectly. This hypothesis was supported by the determined midpoint potential of −285 mV for FdC compared with the lower reduction potential (−490 mV) of FdN.^[^ ^18^ ^]^

We also explored the vastly different structural features of FdC and FdN. We report the X‐ray crystallography structure of FdC, revealing a novel class of [Fe_2_S_2_]‐cluster‐containing Fds essential for N_2_‐fixation under anaerobic photosynthetic conditions. Despite the key structural differences between FdC and FdN, both Fds have their active site FeS‐clusters located very close to the surface (<5 Å). The exposed nature of the active site FeS‐clusters in both FdC and FdN may also account for the degree of functional redundancy between the two Fds. Important for a better understanding of Fds in *R. capsulatus*, our work extends the structural data on FdE associated with FeS‐cluster biogenesis,^[^ ^62^ ^]^ together with the recent data on the thioredoxin‐like [Fe_2_S_2_] ferredoxin linked to cobalamin biosynthesis.^[^ ^63^ ^]^

Overall, we determine that for the electron transfer functions of FdC and FdN, the reduction potential of the FeS‐cluster within an Fd appears to be the most important factor dictating whether an Fd can replace another Fd or not. In the context of nitrogen fixation, it appears that the protein architecture determining the protein interactions is likely not the defining feature in relation to functional specificity. We cannot definitively say why *R. capsulatus* produces both Fds, when FdN can perform both functions, though there are several hypotheses. Firstly, the [Fe_2_S_2_]‐centre of FdC has a higher oxygen stability compared to the two [Fe_4_S_4_]‐clusters in FdN, as highlighted by our two expression systems in *E. coli* (aerobic growth) and *R. capsulatus* (anaerobic growth), respectively. The increased oxygen tolerance of FdC, compared with FdN, is likely important if FprA has a function similar to other oxidases or nitric oxide reductases, as it is known that in the presence of oxygen (O_2_) or nitrous oxide (NO) [Fe_4_S_4_]‐clusters start to rapidly degrade.^[^ ^64^ ^]^ Another hypothesis is that FdC is less wasteful in terms of Fe usage, requiring less Fe atoms per monomer to perform electron transfer, specifically two Fe atoms versus FdN's eight Fe atoms per monomer. Finally, two distinct Fds may facilitate the separation of individual electron transfer pathways, for example nitrogenase metallocluster assembly and electron donation to nitrogenase reductases, since both processes are induced, at the same time, by nitrogen starvation.^[^ ^14^, ^65^ ^]^

This research creates a basis for bioengineering studies of FdC and FdN. Our structure of FdC provides insights into a new class of [Fe_2_S_2_]‐cluster Fds with a novel coordination pattern and structure, allowing for the development of engineered Fds with new structures and electronic properties. Additionally, both FdC and FdN are interesting protein scaffolds due to their small size, the surface‐exposed nature of their FeS‐clusters and their flexible behavior in terms of interacting with a range of partner proteins. Potentially FdC and FdN could be interfaced with a range of proteins and hence could be used for the development of novel electron transport networks.

## 4 Materials and Methods

### 4.1 Chemicals

Unless stated, chemicals were purchased from Carl Roth (Karlsruhe, Germany) or Tokyo Chemical Industry (Tokyo, Japan), enzymes from New England Biolabs (Ipswich, United States), and gases from Air Liquide (Düsseldorf, Germany). DNA primers were purchased from Eurofins (Ebersberg, Germany) and DNA sequencing was performed by Microsynth (Balgach, Switzerland).

### 4.2 Bacterial Strains and Growth Conditions

Constructed strains are listed in Table S1. *R. capsulatus* strains are derived from the strain BS85.^[^ ^66^ ^]^ *R. capsulatus* cells were cultured in PY rich medium or RCV minimal medium as described in *Katzke* et al.^[^ ^67^ ^]^ Unless otherwise specified, RCV minimal media was comprised of 30 mM DL‐Malic Acid, 10 mM serine, 0.2 mM FeSO_4_, 0.8 mM MgSO_4_, 0.7 mM CaCl_2_, 0.05 mM Na_2_EDTA, 0.03 mM thiamine‐HCl, 4.7 mM K_2_HPO4, 5.8 mM KH_2_PO_4_, 45 µM B(OH)_3_, 9.5 µM MnSO_4_, 0.85 µM ZnSO_4_, and 0.15 µM Cu(NO_3_)_2_ set to a pH of 6.8. *Escherichia coli* strains were cultured using Luria‐Bertani rich media (LB), at 37 °C and a shaking speed of 180 rpm, unless otherwise stated.

Photoheterotrophic growth conditions were used to monitor *R. capsulatus* diazotrophic growth. *R. capsulatus* strains were cultured anaerobically in 20 mL vials at 30 °C in RCV minimal media under 1.2 atm nitrogen atmosphere. Cultures were illuminated by LED panels (420 nm, 470 nm blue lights, and 850 nm infrared lights with an output of 12.0 V and light intensity of 80–85 µmol photons m^−2^ s^−1^ per side, or 160–170 total on samples).

### 4.3 Molecular Cloning

Cloning primers and constructed plasmids are listed in Table S1. Both template and constructed plasmids were isolated using GeneJET Plasmid Miniprep Kit (Thermo Scientific, Waltham, United States). *R. capsulatus* genomic DNA was extracted using the Monarch Genomic DNA Purification Kit (New England Biolabs). Polymerase chain reaction (PCR) products were purified using the Monarch PCR & DNA Cleanup Kit (New England Biolabs). Q5 High‐fidelity DNA polymerase was used for PCR reactions.

The N‐terminal tagged *fdxC*‐Strep expression plasmid (pPduP‐*fdxC*‐strep) was constructed using Gibson cloning. PCR was used to amplify both the backbone from plasmid pPduP_Rp_JZ73, using primers pPduP‐F and pPduP‐R, and the insert *fdxC* from *R. capsulatus* BS85, using primers *fdxC*‐strep‐F and *fdxC*‐strep‐R. PCR fragments were assembled using a Gibson assembly following the Gibson Assembly Protocol (E5510) (New England Biolabs). In this construct, the N‐terminal methionine of *R. capsulatus* FdC is replaced by the strep‐tag (MAWSHPQFEK). The C‐terminal tagged *fdxN*‐Strep expression plasmid (pOGG024‐2‐Km^R^ *fdxN‐*strep) was created using Golden Gate cloning. PCR was used to amplify the insert *fdxC*‐strep from BS85 *R. capsulatus*, using primers *fdxN*‐strep‐F and *fdxN*‐strep‐R. PCR fragments were assembled using the NEBridge Golden Gate Assembly Kit (BsaI‐HFv2). In this construct, the strep‐tag (WSHPQFEK) appends the C‐terminal sequence of *R. capsulatus* FdN.

All cloning products were incubated with DpnI overnight at 37 °C. Cloning products were transformed into *E. coli* strain DH5α and single colonies were isolated. Where applicable, *E. coli* colonies were screened using blue‐white screening on LB‐agar plates supplemented with 200 µg/mL XGAL or using colony‐PCR with DreamTaq polymerase 2 × MM. Plasmids were extracted and validated using Sanger sequencing.

### 4.4 *R. capsulatus* Strain Construction

*R. capsulatus* deletion strain construction was conducted using a *sacB* scarless deletion system, as described prior.^[^ ^17^, ^68^ ^]^ In short, deletion plasmids were conjugated into *R. capsulatus*, via diparental mating with ST18 *E. coli* as the donor strain.^[^ ^69^ ^]^ *R. capsulatus* conjugates were passaged, counter‐selected, and sequenced. *R. capsulatus* complementation strains were constructed via diparental mating‐based conjugation, using ST18 *E. coli* as the donor strain.^[^ ^69^ ^]^ ST18 *E. coli* cells were grown in LB media with 50 µg/mL 5‐aminolevulinic acid (C_5_H_9_NO_3_) and 50 µg/mL kanamycin sulfate. *R. capsulatus* cells were grown in PY media and RCV minimal media, where applicable supplemented with 20 µg/mL streptomycin sulfate (Sigma, Darmstadt, Germany) and 50 µg/mL kanamycin sulfate.

### 4.5 Growth Behavior of *R. capsulatus* Complementation Strains

The growth of *R. capsulatus* complementation strains, from three independent cultures per strain, was monitored under photoheterotrophic and N_2_‐fixing conditions (RCV medium with no nitrogen source (no serine) and 1.2 atm nitrogen atmosphere) as previously described.^[^ ^17^ ^]^ OD_660_ of cultures was measured using a TECAN Infinite M Nano+ (TECAN, Männedorf, Switzerland). *R. capsulatus* cultures were supplemented with 50 µg/mL kanamycin sulfate. Doubling times were calculated using an exponential (Malthusian) growth model in GraphPad Prism 10.1.0 for Windows (GraphPad Software, San Diego, USA).

### 4.6 Acetylene Reduction Assay

In vivo Fe‐nitrogenase activity of *R. capsulatus* strains, from three independent cultures per strain, was determined using the acetylene reduction assay as previously described.^[^ ^17^ ^]^ Gas chromatography was used to measure ethylene and ethane, with the PerkinElmer Clarus 690 GC (Perkin Elmer, Waltham, United States). A linear standard curve was used to convert the area into gas amounts. All *R. capsulatus* strains, except those with no net OD_660_ increase (∆*anfDGK*) were normalized by the final OD_660._

### 4.7 FdC Production in *E. coli*

BL21‐Ai cells were transformed with the plasmid pPduP‐*fdxC*‐strep. Transformed BL21‐Ai *E. coli* cells were grown aerobically on solid and in liquid rich media supplemented with 50 µg/mL ampicillin sodium salt (Sigma). A single colony was used to inoculate a pre‐culture of 250 mL which was grown overnight. Using the pre‐culture, 6 L of FdC production main cultures were inoculated to an OD_600_ of 0.05 in LB media supplemented with 150 µM ammonium ferric citrate. FdC main cultures were grown as 750 mL cultures in 1 L shaking flasks. Main cultures were induced at an OD_600_ of 0.4, with 1 mM isopropyl β‐D‐1‐thiogalactopyranoside (IPTG) and 2 mM L‐arabinose. Post‐induction, the cultures were grown overnight with both the shaking speed and temperature reduced to 110 rpm and 30 °C, respectively. To remove residual oxygen prior to harvesting, cultures were transferred to 2 L anaerobically sealed Schott bottles and sparged with argon gas for 30 minutes. All subsequent steps were performed anaerobically. Cultures were harvested, via centrifugation for 15 minutes at 8000 x *g*.

### 4.8 FdN Production in *R. capsulatus*

The *R. capsulatus* FdN‐production strain: ∆*gtaI* ∆*fdxCN fdxN*‐strep was streaked from a glycerol stock on solid PY‐agar and was grown strictly anaerobically and under illumination and an argon atmosphere for two days. To produce a liquid preculture the ∆*gtaI* ∆*fdxCN fdxN*‐strep was grown phototrophically and anaerobically, under illumination by 60‐W krypton lamps (Osram Licht, Munich, Germany), in liquid RCV media supplemented with 50 µg/mL kanamycin sulfate, 3 mM serine as the nitrogen source, 1 mM ferric‐citrate as the iron source and a phosphate buffer concentration of 9.4 mM K_2_HPO_4_, 11.6 mM KH_2_PO_4_. An inoculation loop of cell mass was used to inoculate a 500 mL *R. capsulatus* pre‐culture, which was grown for two days. Using the pre‐culture, 6 L of FdN production main cultures were inoculated to an OD_660_ of 0.05. FdN main cultures were grown for five days (around 110 hours–120 hours) as 750 mL cultures in 1 L Schott bottles under a 1.2 atm nitrogen atmosphere. Cultures were harvested anaerobically, via centrifugation for 60 minutes at 15,970 x *g*.

### 4.9 Protein Purification

All purification steps were performed under strictly anaerobic conditions inside a COY tent under a 95% argon, 5% hydrogen atmosphere (COY Laboratory Products, Grass Lake, United States). All tagged‐protein purifications and size exclusion chromatography analyses were performed using an ӒKTA pure chromatography system (Cytiva, Marlborough, United States). Cell pellets of protein production cultures were isolated by pouring‐off and discarding the liquid supernatants. Pellets were combined and re‐suspended in high‐salt buffer (50 mM Tris, 500 mM NaCl, 10% glycerol, pH 7.8) via agitation with a vortex. Prior to subsequent purification or pellet storage at ‐20 °C, one cOmplete EDTA‐free protease inhibitor tablet (Roche, Basel, Switzerland) and a spatula tip of bovine pancreatic deoxyribonuclease I (Roche) was added. Cells were lysed via three passages through a French‐Press cell disruptor (model FA‐078AE) (Thermo Fisher Scientific, Waltham, United States) at 20,000 p.s.i. Cell lysate was isolated, from the membranes and cell debris, via centrifugation at 130,000 x *g* for 60 minutes at 4 °C and was filtered (0.2 µM pores).

Cell lysates were loaded on to a high‐salt buffer equilibrated 5 mL Strep‐TactinXT 4Flow high capacity FPLC column (IBA Lifesciences, Göttingen, Germany), next the column was thoroughly washed with 20 column volumes of high‐salt buffer. For protein elution, high‐salt buffer containing 50 mM biotin was applied to the column. 1 mL elution fractions were collected and those with significant absorbance at 280 nm were pooled and concentrated to a volume of < 2.5 mL using a high‐salt equilibrated 15 mL Amicon Ultra Centrifugal Filter, 3 kDa MWCO (Merck, Darmstadt, Germany). Concentrated elution was desalted via loading on a low‐salt buffer, 50 mM Tris, 150 mM NaCl, 10% glycerol, pH 7.8, equilibrated disposable PD‐10 desalting column, with Sephadex G‐25 resin (Cytiva). Desalted elutions were concentrated to a volume of <1 mL using a low‐salt equilibrated 15 mL Amicon Ultra Centrifugal Filter, 3 kDa MWCO (Merck). Purified proteins were frozen in liquid nitrogen.

### 4.10 Protein Yield Quantification

The protein yield for FdC was determined using the Quick Start Bradford 1x Dye Reagent (Bio‐Rad Laboratories, Hercules, United States). The protein yield for FdN was determined using the Pierce BCA Protein Assay Kit (Thermo Scientific). All protein quantification assays were performed as 250 µL microplate assays following the manufacturer's instructions in a 96‐well plate (Sarstedt AG & Co. KG, Nümbrecht, Germany) and measured using a TECAN Infinite M Nano+ (TECAN, Männedorf, Switzerland). Protein standards used were Pierce Bovine Serum Albumin Standard Pre‐Diluted Set (Thermo Scientific).

### 4.11 Size Exclusion Chromatography

Purified proteins were concentrated to a volume of <500 µL using an Amicon Ultra Centrifuge‐Filter 3 kDa MWCO (Merck). Proteins were then injected onto a low‐salt buffer (50 mM Tris, 150 mM NaCl, 10% glycerol, pH 7.8) equilibrated Superdex75 10/300 GL column (Cytiva). Equilibration of the SEC column was performed using “Mix A” from the LMW Gel Filtration Calibration Kit (Cytiva). Blue Dextran 2000 was used to determine the void volume. Elution volumes were converted to determined molecular weights (MWs) using a standard curve.

### 4.12 SDS‐Page Analysis

Samples were denatured by boiling in combination with Pierce Lane Marker Reducing Sample Buffer (Thermo Fisher Scientific) at 98 °C for 5 minutes. Samples were then centrifuged at 16,000 x *g* and loaded on a 4–15% Mini‐PROTEAN TGX Precast Protein Gel (Bio‐Rad Laboratories) with the ladder: SeeBlue Plus2 pre‐stained protein standard (Invitrogen, Thermo Fisher scientific, Waltham, United States). Electrophoresis was run for 1 hours at 100 V prior to gel staining with GelCode Blue Safe Protein Stain (Thermo Fisher scientific).

### 4.13 Western Blot Analysis

Protein sample denaturation and electrophoresis was performed as described for SDS‐Page analysis. Post electrophoresis, the gel was washed 3x for 5 minutes in ultrapure water and equilibrated for 10 minutes in Turbo Transfer Buffer (300 mM Tris, 300 mM glycine, 0.05% SDS (m/v), pH 9.0). Proteins were transferred onto a Roti NC transfer membrane using the Trans‐Blot Turbo Transfer System (Bio‐Rad Laboratories). A mixed molecular weight (MW) program was used for the transfer: 7 minutes, 1.3 A, 25 V. The membrane was blocked by incubation with 50 mL 5% (m/v) milk in TBS‐T buffer (10 mM Tris, 250 mM NaCl, 0.05% (v/v) Tween 20, pH 9.0) for 1 hours at 4 °C. The membrane was incubated with Streptag II Antibody HRP Conjugate (Sigma) in 10 mL 5% (m/v) Milk TBS‐T overnight at 4 °C. The membrane was washed 5x for 10 minutes with TBS‐T solution. The membrane was developed using the Amersham ECL Primer Western Blot Kit (Cytiva) and imaged using a ChemiDoc Imaging System (Bio‐Rad Laboratories).

### 4.14 UV‐Vis Spectrophotometry

Spectra in the range of 200 nm to 800 nm were collected using a spectrophotometer (CARY 60, Agilent, Santa Clara, United States). Samples were prepared in an anaerobic tent (COY Laboratory Products) under a 95% argon, 5% hydrogen atmosphere. 1 mL of a 30 µM solution of protein was prepared in 50 mM Tris‐HCl buffer, pH 7.8, in an anaerobic cuvette with a rubber stopper (Starna GmbH, Pfungstadt, Germany). Reduced spectra were recorded after the addition two equivalents (60 nmol) of Na_2_S_2_O_4_ (Fischer Scientific, Waltham, United States). Oxidized spectra were recorded after exposing the reduced sample to air for 240 s.^[^ ^70^ ^]^

### 4.15 Protein Crystallization and X‐ray Analysis

FdC purifications for crystallization were conducted as described above, except with glycerol omitted from all purification buffers. FdC for crystallography was stored anaerobically overnight at 4 °C. FdC was concentrated to a concentration of 5 mg/mL using an Amicon Ultra Centrifuge‐Filter 3 kDa MWCO (Merck). All protein crystallization was conducted in anaerobic conditions under a 95% nitrogen, 5% hydrogen atmosphere (COY Laboratory Products). On sitting‐drop vapour‐diffusion crystallization plates, 0.2 µL of 5 mg/mL FdC was combined with 0.2 µL of crystallization reagent. Plates were regularly screened and promising crystals appeared between 6–12 days. For PDB (9I2A), crystallization occurred in the condition with 0.1 M Tris, pH 8.0, and 20% w/v SOKALAN PA 25 CL. Crystals were cryoprotected ad 40% PEG 400, looped, and frozen liquid nitrogen. X‐ray diffraction data were collected at the beamline ID30B of the European Synchrotron Radiation Facility (ESRF).

The dataset was processed with the XDS software package.^[^ ^71^ ^]^ The structures were solved by molecular replacement using the alpha‐fold model of RcFdC (AF‐D5ARY7‐F1). Molecular replacement was carried out using Phaser of the Phenix software package and refined with Phenix Refine.^[^ ^72^ ^]^ Additional modeling, manual refining, and ligand fitting were done in COOT.^[^ ^73^ ^]^ Final positional and B‐factor refinements as well as water‐picking for the structure were performed using Phenix.Refine. The structure model was deposited at the PDB in Europe under PDB ID: 9I2A. Data collection and refinement statistics are provided in Table S3.

### 4.16 Electron Paramagnetic Resonance Spectroscopy (EPR)

EPR samples were prepared under anaerobic conditions inside an anaerobic glove box under a 95% nitrogen, 5% hydrogen atmosphere (COY Laboratory Products).

The redox titration of as‐isolated FdC was performed in the presence of a 40 µM redox mediator mix in 100 mM Tris, 150 mM NaCl, 10% glycerol, pH 7.8, through the addition of small volumes of 1.56–200 mM sodium dithionite stock solution. The mixture consisted of the following redox mediators: 5,5′‐indigodisulfonate (IDS, E_0_ = ‐125 mV), 2‐hydroxy‐1,4‐napthaquinone (E_0_ = ‐152 mV), sodium anthraquinone 2‐sulfonate (E_0_ = ‐225 mV), phenosafranin (E_0_ = ‐252 mV), safranin O (E_0_ = ‐289 mV), neutral red (E_0_ = ‐329 mV), benzyl viologen (E_0_ = −358  mV) and methyl viologen (E_0_ = ‐449 mV). The concentration of FdC used was 100 µM. Upon reaching the desired redox potential, 300 µl of sample were transferred to an EPR quartz tube, capped, and shock frozen in liquid nitrogen. The InLab Argenthal microelectrode (Ag/AgCl, E = +207 mV vs. H_2_/H^+^, with combined Pt counter electrode) was calibrated with quinhydrone‐saturated pH 4, pH 7, and pH 9 buffer solutions (E = +464.2 mV, E = +291.1 mV, and E = +170.1 mV vs. H_2_/H^+^ at 25 °C) and values were corrected according to a quinhydrone‐saturated pH 7.8 solution (E = +245.5 mV vs. H_2_/H^+^ at 25 °C).

Reduced samples of FdN and FdC were also prepared without the mediator mix in the following buffers: low‐salt buffer for FdC (pH 7.8, 100 mM Tris, 150 mM NaCl, 10% glycerol, 2 mM sodium dithionite) and pH 9.0 buffer for FdN (pH 9.0, 100 mM Tris, 5 mM sodium dithionite). As initial experiments at lower pH did not lead to reduction of FdN, pH 9.0 was used to reduce the FdN sample as a high pH decreases the potential of dithionite.^[^ ^41^, ^43^ ^]^ The concentration of protein used were: 50 µM for FdC and 200 µM for FdN.

Cw‐EPR spectra were recorded with a commercial Bruker spectrometer composed of an X‐band E580‐10/12 bridge and a 4122HQE resonator in perpendicular mode (Bruker, Ettlingen, Germany). The system was equipped with an Oxford Instruments temperature controller and ESR 900 cryostat, which was cryocooled by a Stinger (Cold Edge Technologies, Allentown, Pennsylvania, USA) linked to helium compressor (Sumitomo F‐70, Sumitomo (SHI) Cryogenics of Europe, GmbH, Darmstadt, Germany).

### 4.17 Bioinformatics

Computed molecular weight values of protein sequences were calculated using the ProtParam tool from Expasy.^[^ ^74^ ^]^ Coulombic electrostatic potentials of protein models were determined using ChimeraX.^[^ ^75^ ^]^ Protein models were visualized and analyzed, including root mean square deviation (RMSD) values between Cα atoms, bond length determination, and bond angle values, using PyMOL.^[^ ^52^ ^]^

## Conflict of Interests

The authors declare no conflicts of interest.

## Supporting information



Supporting information

## Data Availability

The data that support the findings of this study are openly available in Edmond ‐ the Open Research Data Repository of the Max Planck Society at https://doi.org/10.17617/3.ASKVZU, reference number [1].
