# Supplementary material for: Two Key Ferredoxins for Nitrogen Fixation Have Different Specificities and Biophysical Properties
Source: Chemistry. 2025 May 30;31(37):e202500844. doi: 10.1002/chem.202500844 (PMC12223475; doi:10.1002/chem.202500844)
Supplement: Supplementary file 1 — Supporting information [file CHEM-31-e202500844-s001.pdf]

Two Key Ferredoxins for Nitrogen Fixation Have Different Specificities and Biophysical Properties

Holly Addison[1], Pascal Pfister[2], Ana Lago-Maciel[1], Tobias J. Erb[2, 4], Antonio J. Pierik[3], Johannes G. Rebelein[1, 4]

1. Microbial Metalloenzymes Research Group, Max Planck Institute for Terrestrial Microbiology, 35043 Marburg, Germany.
2. Biochemistry and Synthetic Metabolism Research Group, Max Planck Institute for Terrestrial Microbiology, 35043 Marburg, Germany
3. Biochemistry, Department of Chemistry, RPTU Kaiserslautern-Landau, 67663 Kaiserslautern, Germany
4. Centre for Synthetic Microbiology (SYNMIKRO), Philipps University Marburg, 35043 Marburg, Germany.

# Correspondence to Antonio J. Pierik, pierik@rptu.de and Johannes G. Rebelein, johannes.rebelein@mpi-marburg.mpg.de.

Supplementary materials:

Table S1: Strains, plasmids and primers used in this study.

Strains:

Strain	Genotype	Reference
R. capsulatus **strains**		
BS85	Δ*nifD*::Sp mutant (Δ*nifDK*)	[1]
BS85 Δ*modABC*	Wild-type: Δ*nifD*::Sp mutant (Δ*nifDK*) Δ*modABC*	[2]
Δ*gtaI*	Δ*nifD*::Sp mutant (Δ*nifDK*) Δ*modABC* Δ*gtaI*	This study
Δ*gtaI* Δ*fdxCN*	Δ*nifD*::Sp mutant (Δ*nifDK*) Δ*modABC* Δ*gtaI* Δ*fdxCN*	This study
Δ*fdxC*	Δ*nifD*::Sp mutant (Δ*nifDK*) Δ*modABC* Δ*fdxC*	[2]
Δ*fdxN*	Δ*nifD*::Sp mutant (Δ*nifDK*) Δ*modABC* Δ*fdxN*	[2]
Δ*anfDGK*	Δ*nifD*::Sp mutant (Δ*nifDK*) Δ*modABC* Δ*anfDGK*::*GmR*	[2]
E. coli **strains**		
DH5α	Φ80*lac*ZΔM15 Δ(*lac*ZYA-*arg*F) U169 *rec*A1 *end*A1 *hsd*R17(r_k^-, m_k^+) *pho*A *sup*E44 *thi*-1 *gyr*A96 *rel*A1 λ^-	Thermo Fisher Scientific Inc. (Waltham, USA) catalogue #18265017
ST18	RP4-2 *Tc*::*Mu Km*::*Tn7* Δ*hemA* mutant	[3]
BL21-AI	F^-*omp*T *hsd*S_B (r_B^-, m_B^-) *gal dcm ara*B::T7RNAP-*tet*A	Thermo Fisher Scientific Inc. (Waltham, USA) catalogue #C607003

Plasmids:

Plasmid	Relevant Features	Reference
pK18mobSacB-Δ*gtaI*	Suicide plasmid, *sacB*, *oriT* (mobilisable), in-frame Δ*gtaI*, Km^R	[4]
pK18mob-SacB2-Δ*fdxCN*	Suicide plasmid, *sacB*, *oriT* (mobilisable), in-frame Δ*fdxCN*, Km^R	[2]
pOGG024-2-Km^R	Broad-host range plasmid, pBBR1 with *oriT* (mobilisable), *lacZα* cassette for golden gate cloning (BsaI), *anfH* promoter, Km^R	[2]
pOGG024-2-Km^R *fdxN*-strep	Broad-host range plasmid, pBBR1 with *oriT* (mobilisable), *fdxN*-strep-tagged (C-terminus) cloned into BsaI site, *anfH* promoter, Km^R	This study
pNMS16-3-*fdxN*	Broad-host range plasmid, repABC4 with *oriT* (mobilisable), *fdxN* cloned into MCS, *anfH* promoter, Km^R	[2]
pNMS16-3-*fdxC*	Broad-host range plasmid, repABC4 with *oriT* (mobilisable), *fdxC* cloned into MCS, *anfH* promoter, Km^R	[2]
pPduP_Rp_JZ73	Inducible *E. coli* expression plasmid, *LacI,* AldDH-strep-tagged (N-terminus), T7 promoter, Am^R	[5]
pPduP-*fdxC*-strep	Inducible *E. coli* expression plasmid*, LacI, fdxC*-strep-tagged (N-terminus), Am^R	This study

Primers:

Primer	Sequence (5'-3')	Purpose
fdxN-Go-R	GATCTAGGTCTCCTATGGCCATGAAGATCGATCCCGA	Amplify *fdxN* for GoldenGate into pOGG024-2-Km^R
fdxN-strep-F	GATCTAGGTCTCTAGCGTTATTTTTCGAACTGCGGGTGGCTCCACGCCGCCGGGTTGATG	Amplify *fdxN* with a C-terminal strep-tag for GoldenGate into pOGG024-2-Km^R

pPduP-F	ATTCATAATCTTTCTAGAAGATCTCCTACAATA	Amplification of pPduP_Rp_JZ73 for Gibson cloning
pPduP-R	CTTCTCGAACTGCGGGTGG	Amplification of pPduP_Rp_JZ73 for Gibson cloning
fdxC-strep-F	CTTCTAGAAAGATTATGAATTCAGGCGGGGCGAACC	Amplification of *fdxC* for Gibson cloning into pPduP_Rp_JZ73
fdxC-strep-R	CCACCCGCAGTTCGAGAAGGACAAGGCCACACTGACGTT	Amplification of *fdxC* for Gibson cloning into pPduP_Rp_JZ73

Table S2: Computationally determined physical and chemical parameters for the tagged *R. capsulatus* proteins FdC and FdN using the ProtParam tool[6].

Protein name	No. of amino acids	Molecular weight (Da)	Extinction coefficient ($M^{-1}cm^{-1}$ at 280 nm)
FdC	104	11273.86	6990
FdN	73	7904.00	6990

Extinction coefficient calculated assuming all Cys residues are reduced.

Table S3: Data collection and refinement statistics. Statistics for the highest-resolution shell are shown in parentheses.

	***Rc*FdC**
**PDB ID**	9I2A
**Wavelength (Å)**	0.87313
**Resolution range (Å)**	19.34 - 1.7 (1.74 - 1.7)
**Space group**	$P\ 2_1\ 2_1\ 2_1$

**Unit cell dimensions**	
***a*, *b*, *c* (Å)**	45.9, 45.8, 85.3
**α, β, γ (°)**	90.0, 90.0, 90.0
**Total reflections**	253927 (35910)
**Unique reflections**	20380 (1387)
**Multiplicity**	12.4
**Completeness (%)**	99.96 (99.64)
**Mean *I*/ σ(*I*)**	17.1 (5.0)
**Wilson *B*-factor**	19.59
**R_{merge}**	0.094 (0.502)
**R_{meas}**	0.099 (0.523)
**R_{pim}**	0.028 (0.147)
**$CC_{1/2}$**	0.999 (0.934)
**Reflections used in refinement**	20380 (1387)
**Reflections used for R-free**	1988 (139)
**R_{work}**	0.2796 (0.3418)
**R_{free}**	0.2838 (0.2881)
**Number of non-hydrogen atoms**	1528
**Macromolecules**	1419
**Ligands**	8
**Solvent**	101
**Protein residues**	191
**RMS (bonds) (Å)**	0.004

**RMS (angles) (°)**	0.55
**Ramachandran**	
**Favoured (%)**	98.40
**Allowed (%)**	1.60
**Outliers (%)**	0.00
**Rotamer outliers (%)**	0.00
**Clashscore**	2.11
**Average *B*-factor Å^2**	21.68
**Macromolecules**	21.48
**Ligands**	21.84
**Solvent**	24.51

Table S4: Bonds lengths within the [Fe_2S_2] cluster of *R. capsulatus* FdC

Bond	Fe_1-S^{γ}38	Fe_1-S^{γ}43	Fe_2-S^{γ}46	Fe_2-S^{γ}81	Fe_1-S_1	Fe_1-S_2	Fe_2-S_1	Fe_2-S_2
**Distance (Å)**	2.34	2.27	2.32	2.29	2.20	2.21	2.20	2.20

Distances are shown correct to two decimal places. Distances were calculated using ChimeraX[7].

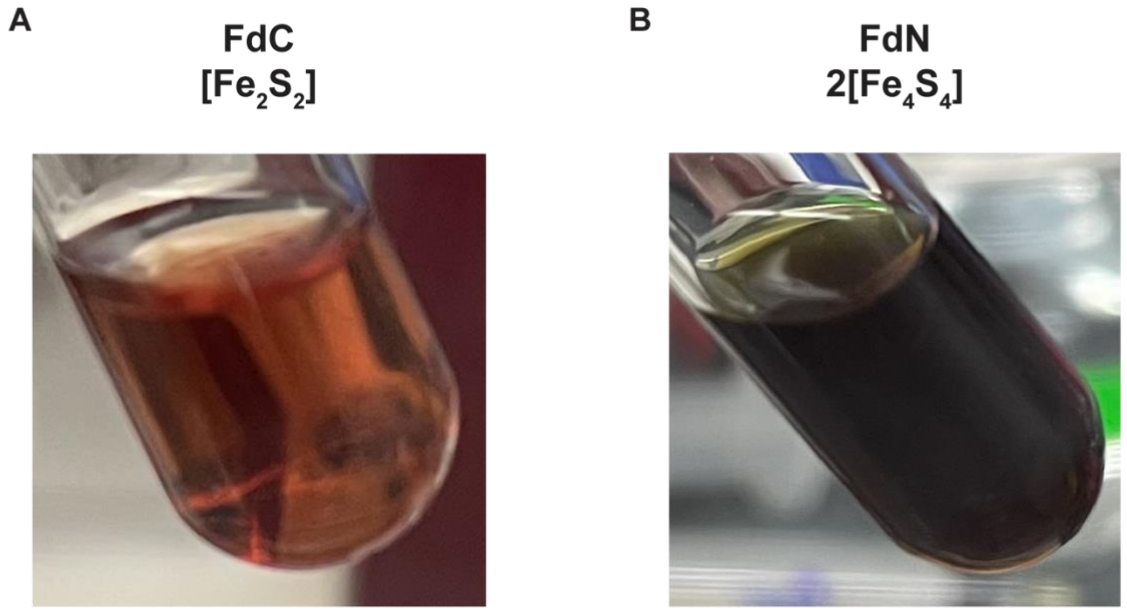

Fig. S1 FdC and FdN elution fractions have strong colours, corresponding to their respective FeS clusters. (A) FdC containing a $[Fe_2S_2]$-cluster has a dark red colour at a concentration of 5 mg/ml. (**B**) FdN containing two $[Fe_4S_4]$-clusters has a dark brown (almost black) colour at a concentration of 73 mg/ml.

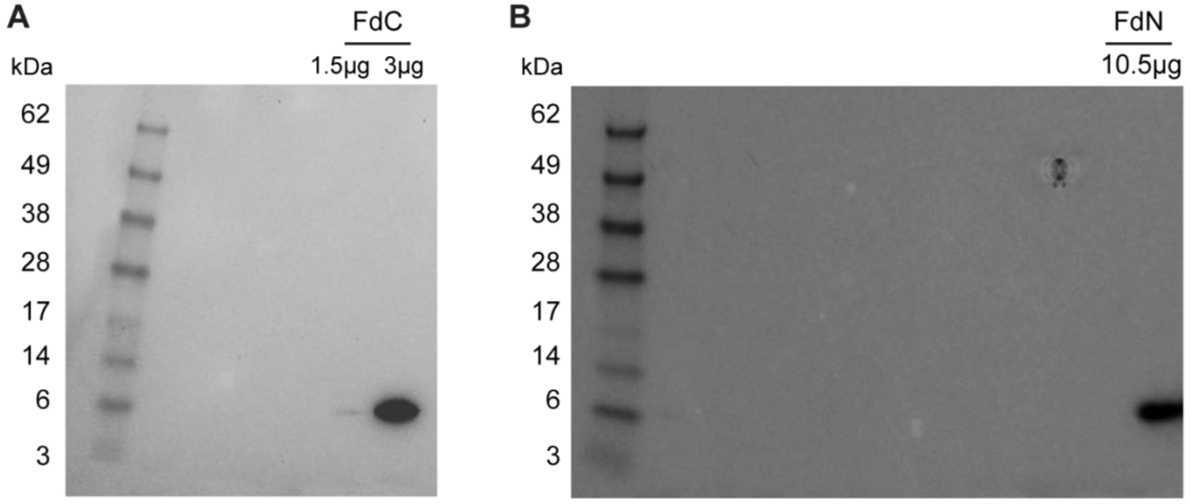

Fig. S2 Uncut α-Strep-HRP western blots of FdC and FdN elution fractions. (A) FdC elution fraction analysed by western blotting with α-Strep-HRP. 1.5µg and 3µg of FdC were loaded as indicated. The band produced by loading 3 µg of FdC is shown in main text Fig. 3B. **(B)** FdN elution fraction analysed by Western blotting with α-Strep-HRP. FdN (10.5 µg) was loaded as indicated. The band produced by loading 10.5µg of FdN is shown in main text Fig. 4B. **(A-B)** Ladder is SeeBlue Plus2 prestained ladder.

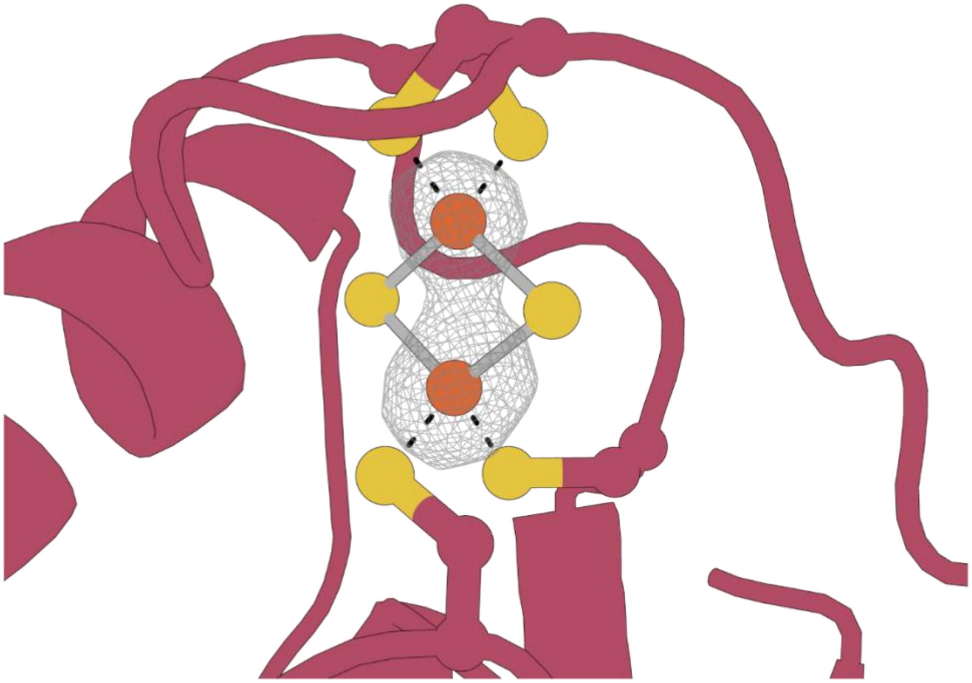

Fig. S3 FdC coordinates one [Fe_2S_2] cluster. Shown is an overlay of the anomalous electron density difference map with the sigma factor (σ) set to 7.0 and the FdC monomer model in a cartoon representation. The FeS cluster and the coordinating Cys residues, C38, C43, C46 and C81, are shown as stick models with iron in orange and sulfur in yellow. Coordinating bonds are shown as dashed lines.

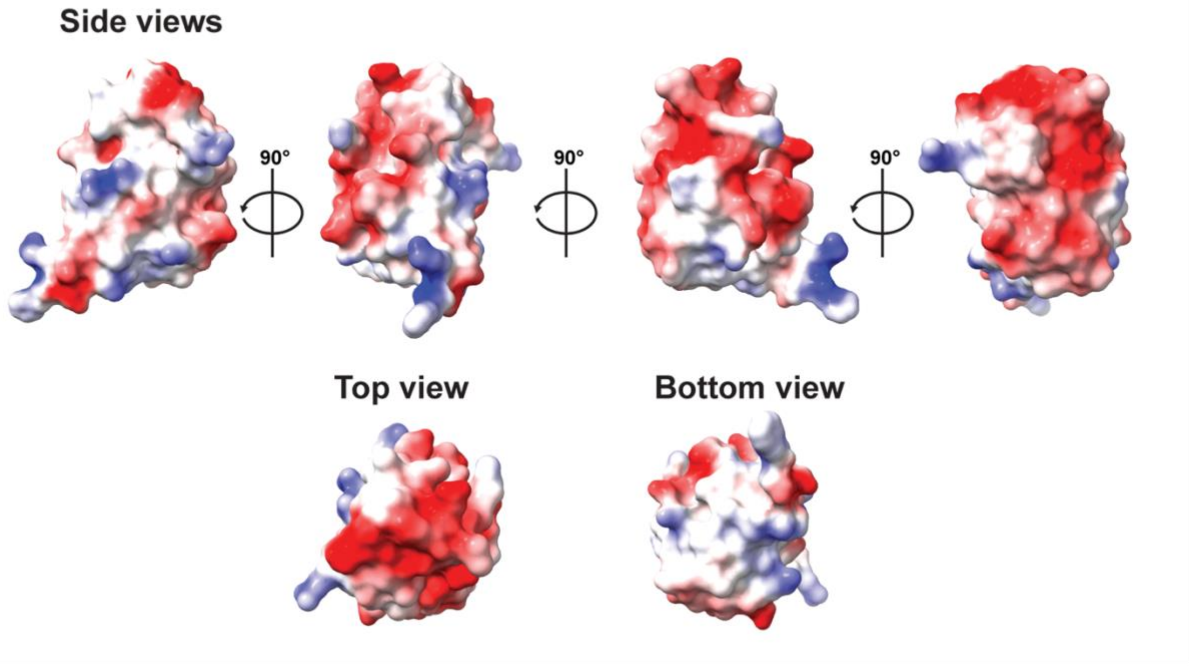

Fig. S4 Electrostatic properties of FdC. Coulombic electrostatic potential calculated in ChimeraX[7]. Surface coloured based on positive potential areas (blue) and negative potential areas (red). **(Top)** All side views of FdC, rotated in 90°C increments. **(Bottom, left)** Top view of FdC. **(Bottom, right)** Bottom view of FdC.

References:

1 Demtröder, L., Pfänder, Y. and Masepohl, B. (2020) *Rhodobacter capsulatus* AnfA is essential for production of Fe-nitrogenase proteins but dispensable for cofactor biosynthesis and electron supply. Microbiology Open. **9**, 1234-1246

2 Addison, H., Glatter, T., Georg, K. A. H. and Rebelein J. G. (2024) Two distinct ferredoxins are essential for nitrogen fixation by the iron nitrogenase in *Rhodobacter capsulatus*. mBio. **15**, e03314-03323

3 Thoma, S. and Schobert, M. (2009) An improved *Escherichia coli* donor strain for diparental mating. FEMS Microbiol Letters. **294**, 127-132

4 Schmidt, F. V., Schulz, L., Zarzycki, J., Oehlmann, N. N., Prinz, S., Erb, T. J. and Rebelein, J. G. (2023) Structural Insights into the Iron Nitrogenase Complex. Nature Structural & Molecular Biology. **31**, 150–158

5 Zarzycki, J., Sutter, M., Cortina, N. S., Erb, T. J. and Kerfeld, C. A. (2017) In Vitro Characterization and Concerted Function of Three Core Enzymes of a Glycyl Radical Enzyme - Associated Bacterial Microcompartment. Scientific Reports. **7**, 42757

6 Gasteiger, E., Hoogland, C., Gattiker, A., Duvaud, S. e., Wilkins, M. R., Appel, R. D. and Bairoch, A. (2005) Protein Identification and Analysis Tools on the ExPASy Server. In The Proteomics Protocols Handbook (Walker, J. M., ed.), pp 571-607, Humana Press, Totowa, NJ

7 Pettersen, E. F., Goddard, T. D., Huang, C. C., Meng, E. C., Couch, G. S., Croll, T. I., Morris, J. H. and Ferrin, T. E. (2021) UCSF ChimeraX: Structure visualization for researchers, educators, and developers. Protein Science. **30**, 70-82
